# Neglected tropical diseases risk correlates with poverty and early ecosystem destruction

**DOI:** 10.1186/s40249-023-01084-1

**Published:** 2023-04-10

**Authors:** Arthur Ramalho Magalhães, Cláudia Torres Codeço, Jens-Christian Svenning, Luis E. Escobar, Paige Van de Vuurst, Thiago Gonçalves-Souza

**Affiliations:** 1 Laboratory of Ecological Synthesis and Biodiversity Conservation (ECOFUN), Federal Rural University of Pernambuco, Recife, PE Brazil; 2 Scientific Computation Program (PROCC), Oswaldo Cruz Foundation (Fiocruz), Rio de Janeiro, RJ Brazil; 3 Center for Ecological Dynamics in a Novel Biosphere (ECONOVO) & Center for Biodiversity Dynamics in a Changing World (BIOCHANGE), Department of Biology., Aarhus University, Aarhus, Denmark; 4 Department of Fish and Wildlife Conservation, Virginia Tech, Blacksburg, VA USA; 5 Center for Emerging Zoonotic and Arthropod-Borne Pathogens, Virginia Tech, Blacksburg, VA USA; 6 Translational Biology, Medicine and Health Program, Virginia Tech Graduate School, Blacksburg, VA USA

**Keywords:** Disease ecology, Ecological niche model, Socioecological system, Vector-borne diseases, Zoonosis, Brazil

## Abstract

**Background:**

Neglected tropical diseases affect the most vulnerable populations and cause chronic and debilitating disorders. Socioeconomic vulnerability is a well-known and important determinant of neglected tropical diseases. For example, poverty and sanitation could influence parasite transmission. Nevertheless, the quantitative impact of socioeconomic conditions on disease transmission risk remains poorly explored.

**Methods:**

This study investigated the role of socioeconomic variables in the predictive capacity of risk models of neglected tropical zoonoses using a decade of epidemiological data (2007–2018) from Brazil. Vector-borne diseases investigated in this study included dengue, malaria, Chagas disease, leishmaniasis, and Brazilian spotted fever, while directly-transmitted zoonotic diseases included schistosomiasis, leptospirosis, and hantaviruses. Environmental and socioeconomic predictors were combined with infectious disease data to build environmental and socioenvironmental sets of ecological niche models and their performances were compared.

**Results:**

Socioeconomic variables were found to be as important as environmental variables in influencing the estimated likelihood of disease transmission across large spatial scales. The combination of socioeconomic and environmental variables improved overall model accuracy (or predictive power) by 10% on average (*P* < 0.01), reaching a maximum of 18% in the case of dengue fever. Gross domestic product was the most important socioeconomic variable (37% relative variable importance, all individual models exhibited *P* < 0.00), showing a decreasing relationship with disease indicating poverty as a major factor for disease transmission. Loss of natural vegetation cover between 2008 and 2018 was the most important environmental variable (42% relative variable importance, *P* < 0.05) among environmental models, exhibiting a decreasing relationship with disease probability, showing that these diseases are especially prevalent in areas where natural ecosystem destruction is on its initial stages and lower when ecosystem destruction is on more advanced stages.

**Conclusions:**

Destruction of natural ecosystems coupled with low income explain macro-scale neglected tropical and zoonotic disease probability in Brazil. Addition of socioeconomic variables improves transmission risk forecasts on tandem with environmental variables. Our results highlight that to efficiently address neglected tropical diseases, public health strategies must target both reduction of poverty and cessation of destruction of natural forests and savannas.

**Supplementary Information:**

The online version contains supplementary material available at 10.1186/s40249-023-01084-1.

## Background

Neglected tropical diseases impacted at least 1.74 billion people globally in 2019 and are associated with significant morbidity and public health burden [1, 2]. One remarkable example is dengue fever, which causes a significant economic impact on governments and households. For instance, a patient may spend 14–18 days in hospital at a cost of USD 514–1500, a severe or unbearable cost for families in poverty without adequate social security [1, 3]. To reduce the risk of occurrence and emergence of infectious diseases, several approaches have been developed [4]. Recent strategies include interdisciplinary and multisectoral collaboration between public health and governmental institutions. For instance, the One Health approach proposes the monitoring of disease transmission between people, animal vectors, and reservoirs, as well as the ecosystem, and encompasses the agriculture, health, and environment sectors of society [5]. The Global Health Security Agenda [6] seeks to strengthen countries’ capacity for the prevention and surveillance of infectious diseases. These strategies are consistent with the third Sustainable Development Goal of the United Nations which is to ensure healthy lives and well-being (https://www.undp.org/sustainable-development-goals). Global health security is a goal that is conditional upon adequate infectious disease monitoring and spatial risk assessment [6, 7]. Hence, prevention and monitoring are necessary strategies to ensure global health security, and are a common focus for sustainable development achievement efforts [5, 6, 8].

**Fig. 1:**
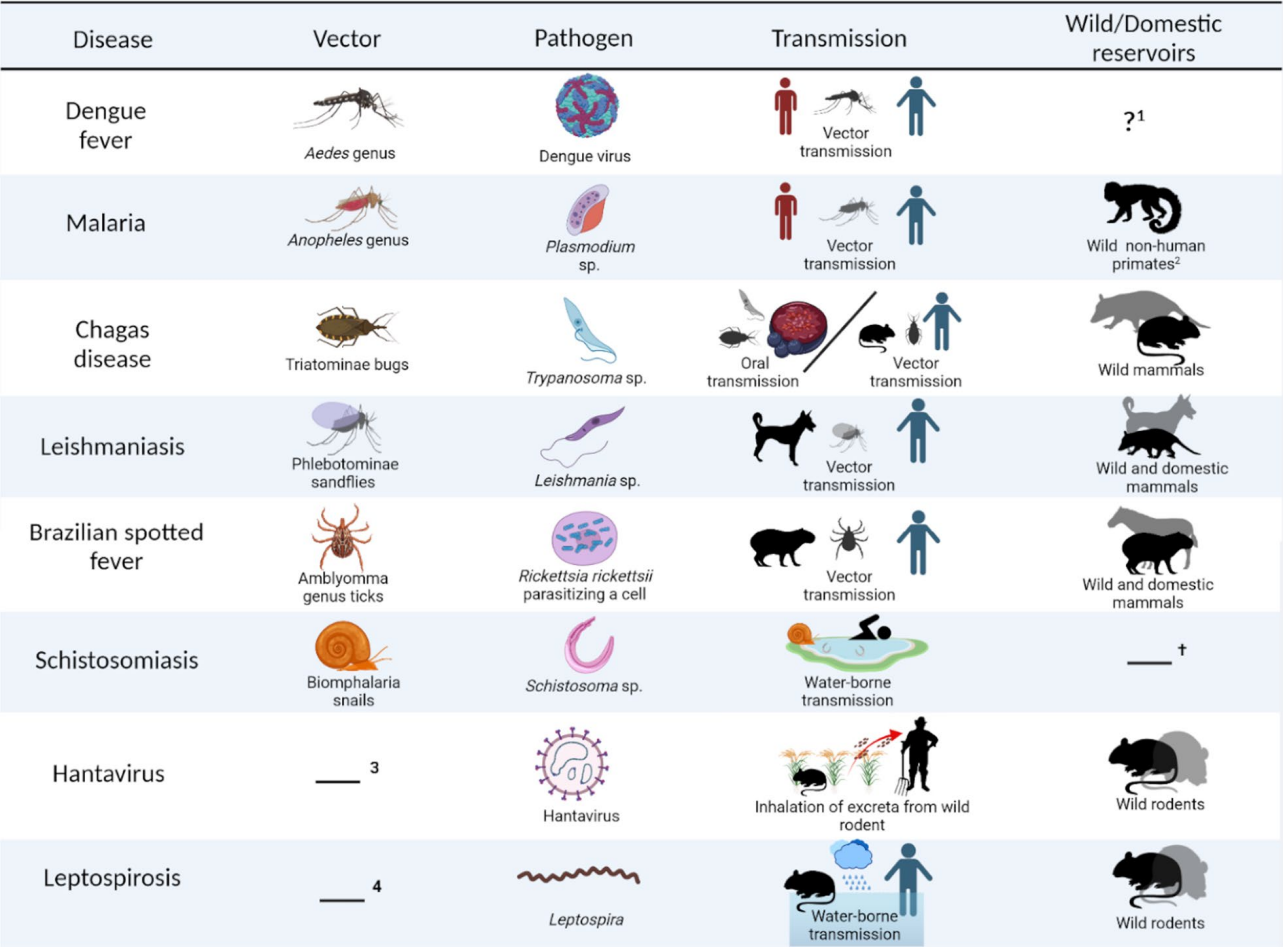
Main reservoir, pathogen, mode of transmission of diseases included in the study. Dengue fever is transmitted by mosquitos of the *Aedes* genus. Transmission happens when an infected person is bitten by mosquitoes which then transfer the pathogen to a new host. *Anopheles* mosquitoes transmit malaria from an infected individual to a new host. *Plasmodium vivax* is the primary pathogen responsible for malaria in Brazil [13, 67]. Chagas disease is caused by protozoa from the *Trypanosoma* genus. Chagas disease transmission occurs through the bite of hematophagous Triatominae bugs or orally by the ingestion of contaminated food (e.g., açaí [13]). Cutaneous and visceral leishmaniasis are mainly transmitted by the bite of Phlebotominae sandflies and infect a wide range of hosts capable of infecting domestic and wild mammals. Brazilian spotted fever is primarily transmitted by the bite of ticks from the *Amblyomma* genus and is brought by *Rickettsia* bacteria. Additionally, it has a wide range of reservoirs including capybaras and horses [13]. Schistosomiasis is a helminth-caused disease, transmitted by contact with water where *Biomphalaria* snails are present so the parasite can complete its life cycle to be transmitted. Hantavirus pulmonary syndrome is transmitted by the inhalation of excreta from infected wild or domestic rodents [21]. Leptospirosis is caused by the bacteria *Leptospira*, transmitted by contact with water contaminated with rodents’ urine [13]. Figure made using Biorender.com (2022) and Phylopic (phylopic.org). ^1^While the role of wild reservoirs of dengue virus is not fully elucidated in Brazil or South America it is discussed whether marsupials and bats are potential reservoirs in transmission cycles in the Americas, however there is no evidence of their significance in the cycle of transmission [68]. ^2^Non-human primates can serve as reservoirs for malaria but only in its zoonotic manifestations, which can even have a distinct pathogen, *Plasmodium simium*, and have a distinct cycle from typical malaria, which is caused by *Plasmodium vivax* [67]. ^†^Although marsupials, cattle, and rodents can be infected by *Schistosoma*, there is no proof that these animals serve as significant disease reservoirs [16]. Instead, the maintenance of the schistosomiasis transmission cycle depends heavily on infected humans. ^3^Although some researchers regard the role of wild rodents as vectors of hantavirus pulmonary syndrome transmission, here we will use the definition that wild rodents that are hosts to hantavirus act as reservoirs only. ^4^Although rodents can be considered vectors of the leptospirosis cycle, we considered the concept that domestic rodents are hosts and only reservoirs of leptospirosis

An effective approach of disease surveillance is to investigate the underlying determinants of disease emergence and recurrence in Latin America [4]. The natural history of a disease system is a key aspect to consider when aiming to reconstruct and predict disease outcomes. Heterogeneities and particularities in vector-borne disease cycles are influenced by the environment [9]. For instance, the availability of mosquito breeding sites is a significant factor in the spread of dengue fever and malaria. This includes the former requiring artificial water containers or ponds, as a premise for the spread of *Aedes* mosquitoes, and the latter requiring forest related water bodies in shadowed areas, *Anopheles* mosquitoes most common breeding sites [10, 11]. Dengue virus encompasses serotypes 1 to 4, while malaria is caused by protozoans of the *Plasmodium* genus, mainly by *P. falciparum* and *P. vivax* [1]. Furthermore, Chagas disease can be spread by contact with hematophagous triatomine (kissing bug) feces infected by *Trypanosoma* protozoans [12, 13]. Usually, contamination occurs trough kissing bug bites, or through contaminated food [12, 13]. The presence of the triatomine bugs is associated with poor household construction [1]. Cutaneous, and visceral leishmaniasis are also transmitted by the bites of arthropod vectors (female phlebotomine sandflies) and are caused by protozoans of genus *Leishmania* [1, 13]. Leishmaniasis transmission is associated with forest fragmentation [14]. Moreover, Brazilian spotted fever is caused by bacteria of the genus *Rickettsia*, and is transmitted by tick bites. Transmission usually encompasses an animal reservoir such horses, cattle or capibaras [15] (See Fig. 1). Despite significant progress in understanding the natural history of vector-borne and neglected tropical diseases, the relationship between disease risk and socioeconomic and environmental factors is still poorly understood.

In the case of directly or environmentally transmitted diseases such as schistosomiasis and leptospirosis which are mainly transmitted by contaminated bodies of water, heterogeneities in the environment become direct drivers of transmission [16–18]. Intestinal schistosomiasis is caused by the helminth *Schistosoma mansoni* and its transmission cycle encompasses *Biomphalaria* snails as reservoir hosts [13]. Leptospirosis is caused by bacteria of *Leptospira* genus and its transmission cycle develops in the presence of urine of infected domestic rodents [19]. Likewise, hantavirus is transmitted by inhalation of particles contaminated with the feces of infected wild rodents [20]. Usually, it more heavily affects human in rural environments who work with crops, specifically sugar cane [21]. Hantavirus manifests as hantavirus pulmonary syndrome and can be fatal for infected individuals.

As the environment plays a key role in disease transmission and maintenance, models linking environmental covariates with disease occurrence are often used in modern disease transmission risk mapping [21–26]. Neglected tropical diseases, for example, have strong ties to environmental parameters such as temperature and land cover, which correspond to the biological responses of vector survival, abundance, and pathogen transmission [21]. Precipitation and temperature have been frequently used to forecast the spatiotemporal occurrence of vector-borne diseases and zoonotic diseases like hantavirus [21, 27, 28]. The predictive ability of the climate on disease spatial distribution is linked to the importance of optimal temperatures and water availability for parasite reproduction and survival [22]. Environmental changes, measured as landscape change or ecosystem loss, have been used as predictor variables of multiple aspects of disease in spatiotemporal analysis [29]. When ecosystem loss occur (hereafter referred to as ‘ecosystem destruction’) it may promote disease outbreaks by facilitating the distributional overlap of pathogens, vectors, and reservoirs with domestic animals and humans [30]. In addition, ecosystem destruction can change the abundance of reservoirs and vectors [21], thus augmenting the stability of different host–pathogen systems. In Brazil, current evidence shows that deforestation promotes outbreaks of malaria [31] and others diseases with high morbidity and mortality [1].

Socioeconomic factors also influence how human diseases emerge and persist, since they affect how individuals live, their quality of life, and their interaction with the environment [32, 33]. Neglected tropical diseases, such as Chagas disease, schistosomiasis, dengue, and leishmaniasis are linked to poverty. Neglected tropical diseases disproportionately affect lower income households, resulting in low productivity, a high epidemiological burden (i.e., mortality and morbidity), disability, and social stigma [32, 34]. Poverty, measured as low proportional income, is a key predictor of disease presence and prevalence at individual or spatial level, therefore, it may help explain the spatial distribution of infectious disease [32]. Urbanization facilitates disease transmission, as demonstrated by dengue fever where mosquitoes thrive in human modified environments that offer abundant resources, such as breeding sites [3]. Similarly, a lack of access to clean water and sanitation has been shown to increase the risk of disease transmission for diseases such as schistosomiasis [1]. Interaction between infectious disease and poverty creates a difficult-to-break cycle, or poverty traps, which can last for generations if proper health interventions are not applied, creating an increasing call to properly surveil infectious diseases [6, 32]. Nevertheless, this challenge is exacerbated in low-income regions where these data are most needed, as data are typically incomplete, sparse, or rarely available [33]. As a result, until recently, the use of the socioeconomic components in disease modeling was mostly overlooked, and was not often included in the ecological niche modeling framework of disease risk mapping [22].

The ecological niche modeling framework can be used to delimit the realized ecological niche of parasite species. Realized ecological niches provide proxies of the influence of physiological tolerance, biological interactions, and dispersal on the ecology of organisms (e.g., pathogens, vectors) and are measured as a hypervolume along environmental variables occupied by the species [35]. Here, to elucidate the effects of social variables in forecasting the geographic distribution of disease transmission, we combined socioeconomic and environmental variables to address these gaps and improve the predictability of neglected tropical diseases throughout all Brazilian regions. In addition, we sought to investigate the main socioeconomic and environmental drivers affecting the disease transmission risk. We built ecological niche models using different sets of variables: socioeconomic (e.g., mean income, mean income inequality Gini index, housing quality) and environmental (e.g., temperature, precipitation, ecosystem destruction) variables in combined (composite) and environmental only (simplified) models, using disease occurrence as the response variable. Furthermore, we assessed model predictability (capacity to predict disease occurrence) using different model configurations to tease apart which variables best predict disease occurrence. We modeled five vector-borne (Chagas disease, dengue fever, malaria, leishmaniasis, and Brazilian spotted fever) and three directly (environmentally) transmitted zoonotic (hantavirus, schistosomiasis, and leptospirosis) neglected tropical diseases to determine if model predictability and relevant predictor variable were consistent across infectious diseases of different natural history. We hypothesized that incorporating socioeconomic variables into such predictive models would add more precision in limiting disease risk in areas with high socioeconomic inequality and widespread poverty. Moreover, integrating relevant socioeconomic and environmental variables could be a significant step forward aligning the Global Health Security agenda within the United Nation’s Sustainable Development goals (https://www.undp.org/sustainable-development-goals).

## Methods

### Disease occurrence data

The disease occurrence data (i.e., locations where disease was reported) came from the Brazilian Ministry of Health database (DATASUS [36]). This database contains all the reported cases of diseases that require mandatory notification, such as those of epidemic potential. We collected data of infectious disease from 2007 to 2019, which is the span of time with lab-confirmed occurrences available. Only new occurrences, (i.e., incidence records) were regarded as a result. The chosen diseases were mediated by at least one species of vectors, hosts, or reservoirs, hence being a subject to environmental influence. To reduce uncertainty only laboratory confirmed cases were considered in the study. To avoid interference from vaccination that could potentially bias the models, we excluded diseases that were preventable with high vaccination coverage, such as yellow fever. Eight diseases met these requirements, namely the five vector-borne diseases dengue fever, malaria, Chagas disease, cutaneous and visceral leishmaniasis, and Brazilian spotted fever, as well as three zoonotic diseases including schistosomiasis, leptospirosis, and hantavirus pulmonary syndrome. It is important to keep in mind that the DATASUS disease-occurrence data has several limitations. For instance, the unequal distribution of healthcare coverage in Brazil, especially the diagnostic capacity, could potentially bias the distributions of disease occurrence. Nevertheless, the Brazilian healthcare system is one of the largest integrated healthcare systems in the world and has a reasonable reach capacity, being able to attend isolated populations throughout its territory [37]. While this does not solve biased reporting, it does mitigate potential spatial biases in disease occurrence. Additional file 1: Table S1 expands and explores these and other potential limitations of our method and provides more details on the disease occurrence data (Additional file 1).

The disease occurrences were assigned to the municipality where the infection occurred, as declared in the DATASUS database. Only locality of disease presence was regarded, not the number or prevalence of cases. Exact locations of disease were not available, so they were assigned to their corresponding municipality administrative center (available from IBGE, The Brazilian Institute of Geography and Statistics [38]). Municipalities were unequal in size, so we standardized occurrences inside an 18 km grid, which resembles the cell size of predictor variables (18 km). To mitigates spatial autocorrelation, we used only one presence point per cell of the spatial extent of the study area. Potential consequences of this approach are discussed in Additional file 1.

### Predictor variables

We employed temperature-derived and precipitation-derived variables from WorldClim [39] with a spatial resolution of 10 arc-minutes (approx.18 km) averaged for the period 1970–2000. Worldclim bioclimatic variables are biologically associated to distributional limits of plant and animal species and are often used in ecological niche modeling to estimate environmental limits [39]. For additional landscape-change variables, we used natural habitat cover from the year 2016 (the sum of forest and savannah formation from each municipality in 2016). We used the values from 2016 since it was the median year between occurrence cases. Furthermore, we used ecosystem destruction between 2009 and 2018 (the sum of natural forest and grassland cover that were transformed into urban, pasture or plantation areas) from MapBiomas project to the municipality level (mapbiomas.org) [40].

As socioeconomic variables, we explored proxies for sanitation, demography, and income. As proxies of sanitation, we used percentages of households with piped water, percentages of households with toilets derived from the 2010 Brazilian socioeconomic census [38]. We also used estimates of population density [41], the mean human development index for each municipality [38], and the Gini index from IBGE [38], which is a coefficient of income disparity. All of these socioeconomic variables are associated with disease vulnerability [42]. We also used the per capita gross domestic product (GDP) as a proxy of socioeconomic status and economic development [43, 44]. All socioeconomic and environmental variables were resampled to approx. 18 km (10’) to match the spatial resolution of occurrence and climatic variables, including variables addressed to municipalities’ bounds. GDP and the Worldclim variables values were extracted at the municipality administrative center coordinate. More details on environmental and socioeconomic variables such as original resolution and source are described at Additional file 1: Table S2.

### Variable selection and correlation

To test whether socioeconomic variables improve model performance for each disease, we made two sets of models: one set of models with the environment and socioeconomic variables (combined model) and one set of models with environment-only variables (simple model). In building the models, we started by checking the correlation between all the predictor variables. We allowed the fewest number of variables in each type of model as possible in accordance to the Occam’s razor principle, which prioritizes the simplest explanation. We removed variables with > 70% correlation, aiming to retain the ones with biological linkages with overall disease transmission (Additional file 2). The final set of variables for the combined models included mean annual temperature and annual precipitation, which are linked to vector or reservoir tolerance and suitability [22, 24, 45]; environmental destruction between 2009 and 2018 which was highly correlated with 2016 native forest/vegetation cover (74%), and often associated with pathogen spillover [31, 46, 47]; GDP (highly correlated with human population, 78%), Gini coefficient (proxy for inequality), and proportion of households without a toilet (highly correlated with human development index, − 83%) which are often regarded as proxies of population vulnerability [13, 48]. We used a similar set of variables when building the environmental-only set of variables for the composite model, keeping only the environment-related (temperature, precipitation, loss of natural habitat). Additionally, we used the ‘vifstep’ function from the *sdm* R package [49] to choose a less correlated environmental variable to add to the model, so that both models have a similar number of variables and can be compared (see Additional file 2).

### Ecological niche modeling

Models for each disease were built using four correlative methods (i.e., generalized linear models, Maxent, random forest, and support vector machines) and one climatic envelop method (Bioclim). These algorithms are more conservative algorithms since they prioritize interpolation over extrapolation [50, 51]. Reduced extrapolation is desirable in disease risk mapping to mitigate overprediction in environmental conditions beyond the observed values [51]. We used cross-validation for model calibration using the h-block strategy that generates nine independent models for each algorithm, for a total of 45 models per disease [52]. Then, using the ‘bin model()’ function from the *ntbox* R package, we transformed each model using a 10% presence threshold to binarize a continuous probability map into presence and absence maps [53]. After binarization, we generated ensemble models by summarizing the algorithm models together for each disease. Each model ensemble represented consensus among algorithms on the presence of conditions suitable for long-term disease transmission as a proxy for disease transmission risk [54].

### Model evaluation and test

After building two ensemble models for each disease, we evaluated their quality using a partial receiver operating characteristic metric (pROC) that overcomes the limitation of classical ROC and area under the curve (AUC) approaches [55]. ROC and AUC are classic model evaluating metrics that partially ignore the goodness of fit of the models and are usually biased to favor some algorithms over others. The AUC/ROC metric assume algorithms that span broader predicted areas regardless of commission error as accurate. In other words, the extent of the background area affects its outcomes [55, 56]. The pROC values were developed using the *ntbox* R package [53] and exhibit a range from zero to two, where values above one represent predictions better than random expectations [55]. Finally, to test whether socioeconomic-environmental models performed overall better than environmental-only models in all diseases, we used a simple *t*-test for paired samples using pROC values.

### Relative variable importance and response curves

To explore the relationship between environmental and socioeconomic variables and disease-case occurrence, we used response curves generated by the evaluation strip method [57]. The evaluation strip method addresses the visualization of predicted responses of a species (in this case, a disease, or pathogen) to environmental variables. Response curves and relative variable importance analysis were performed using the *sdm* package [49]. The relative variable importance identifies the most important variables in the model, and response curves inform variable effects in relation to disease occurrences. Both response curves and variable importance were generated based on the ensemble model (averaged by all algorithms) for each variable set (environmental only and socioenvironmental variable set).

## Results

We modeled the spatial distribution of disease-transmission risk for nine neglected tropical diseases in Brazil from 2007 to 2019. In total we assessed 723,109 disease occurrence records. The number of confirmed cases ranged from 219 for Chagas disease to 429,052 cases for dengue fever. Diseases such as leptospirosis, dengue fever, malaria, and leishmaniasis were widespread across the Brazilian territory. Overall, the composite models including both socioeconomic and environmental predictors performed 10% better (based on partial-ROC estimates, α = 0.05; Table 1) than the simple model using only environmental predictors.

**Table 1:** The partial receiver operating characteristic values (pROC) obtained from diseases ensemble models, resulting from socioeconomic (S) and environment only (E) sets of variables

	Socioenvironmental			Environment only			Comparison (S-E)
	Mean AUC	Mean pROC ratio at 5%	*P* value	Mean AUC	Mean pROC ratio at 5%	*P* value	Change in pROC (%)
Hantavirus	0.88	1.56	0.00	0.89	1.43	0.00	9%
Leptospirosis	0.78	1.28	0.00	0.74	1.10	0.00	16%
Schistosomiasis	0.87	1.48	0.00	0.85	1.41	0.00	5.19%
Dengue fever	0.74	1.27	0.00	0.66	1.07	0.00	18.8%
Malaria	0.72	1.17	0.00	0.56	1.03	0.02	14%
Acute Chagas disease	0.89	1.37	0.00	0.82	1.35	0.00	1.73%
Leish. Visceral	0.80	1.34	0.00	0.77	1.31	0.00	2.1%
Leish. Cutaneous	0.71	1.23	0.00	0.64	1.08	0.00	13.1%
Brazilian spotted fever	0.95	1.72	0.00	0.94	1.74	0.00	***−1.1%***
Average	–	1.40	–	–	1.30	–	**10%**

All models performed better than random. The bold italic value symbolizes the only disease, Brazilian spotted fever, in which the addition of socioeconomic variables in the model lowered model predictability

pROC: Partial receiver operating characteristic; AUC: Area under the curve metric; S: Models made socioenvironmental set of variables; E: Models made with environmental set of variables; –: Not applicable

### Socioeconomic variables

**Fig. 2:**
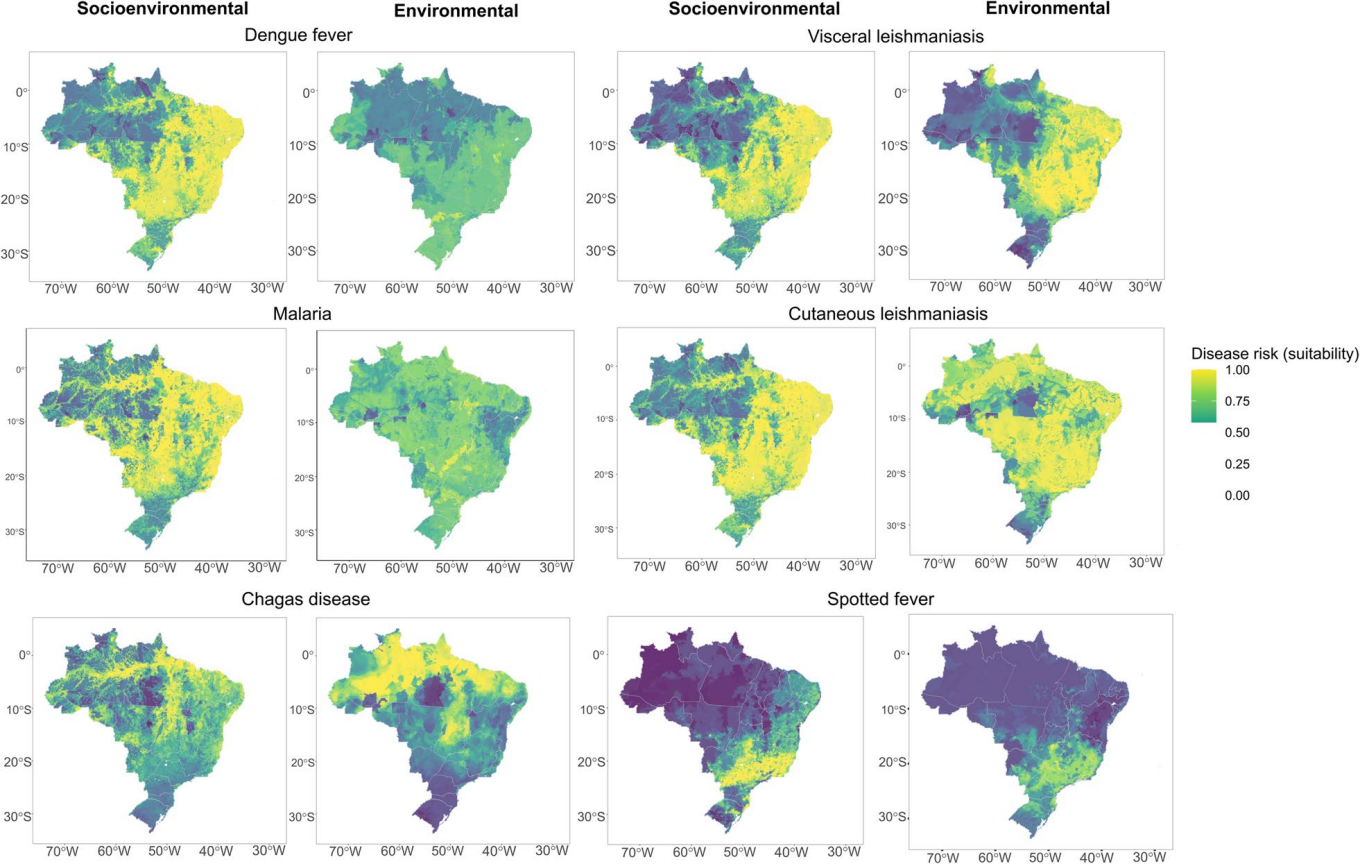
Ensemble suitability models for vector-borne diseases made with socioenvironmental and environmental predictors models (composite socioenvironmental models and simple environmental models). Dark blue indicates low disease suitability, while light yellow indicates high disease suitability to presence or risk

**Fig. 3:**
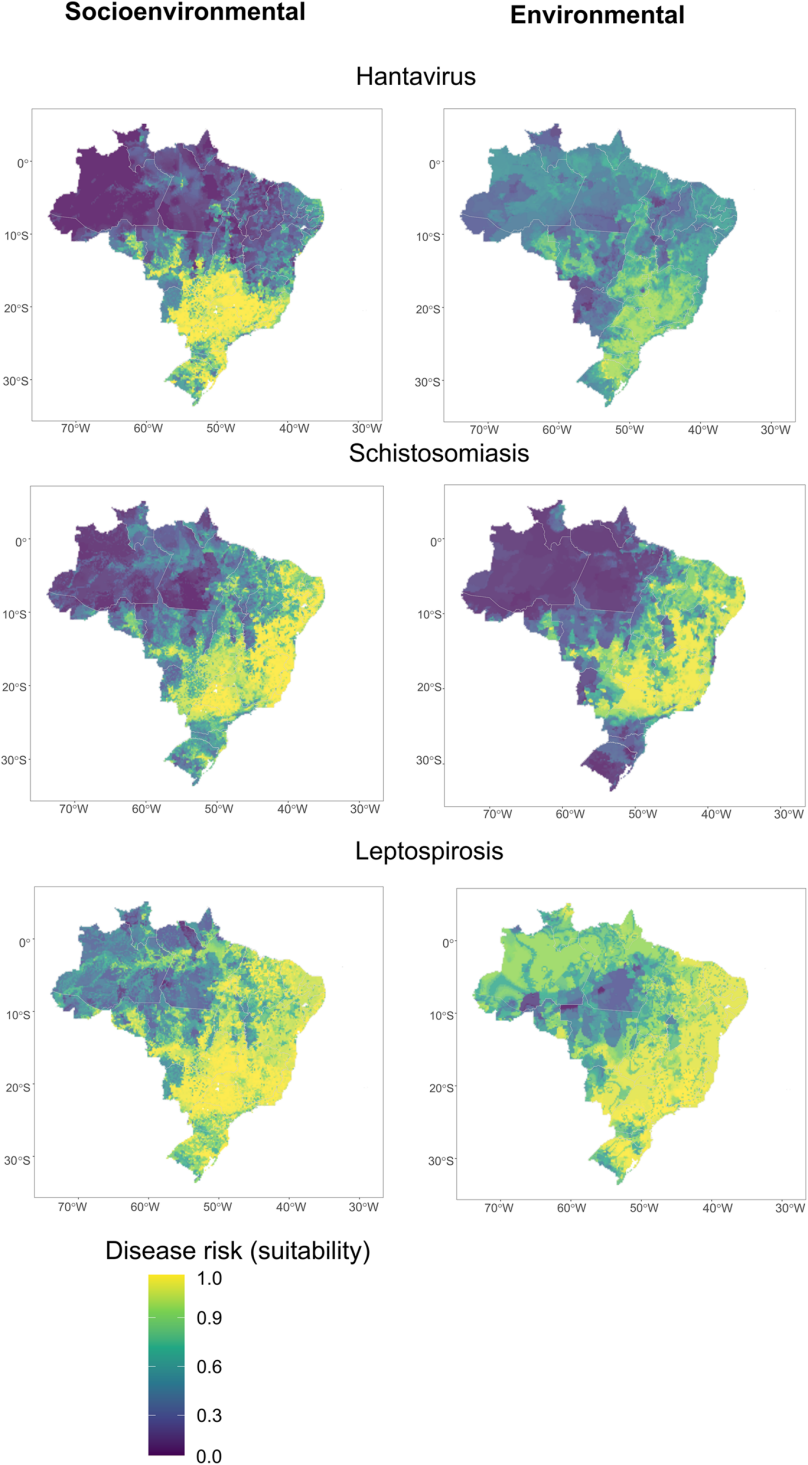
Ensemble suitability models for zoonotic diseases made with socioenvironmental and environmental predictors models (composite socioenvironmental models and simple environmental models). Dark blue indicates low disease suitability, while light yellow indicates high disease suitability to presence or risk

**Fig. 4:**
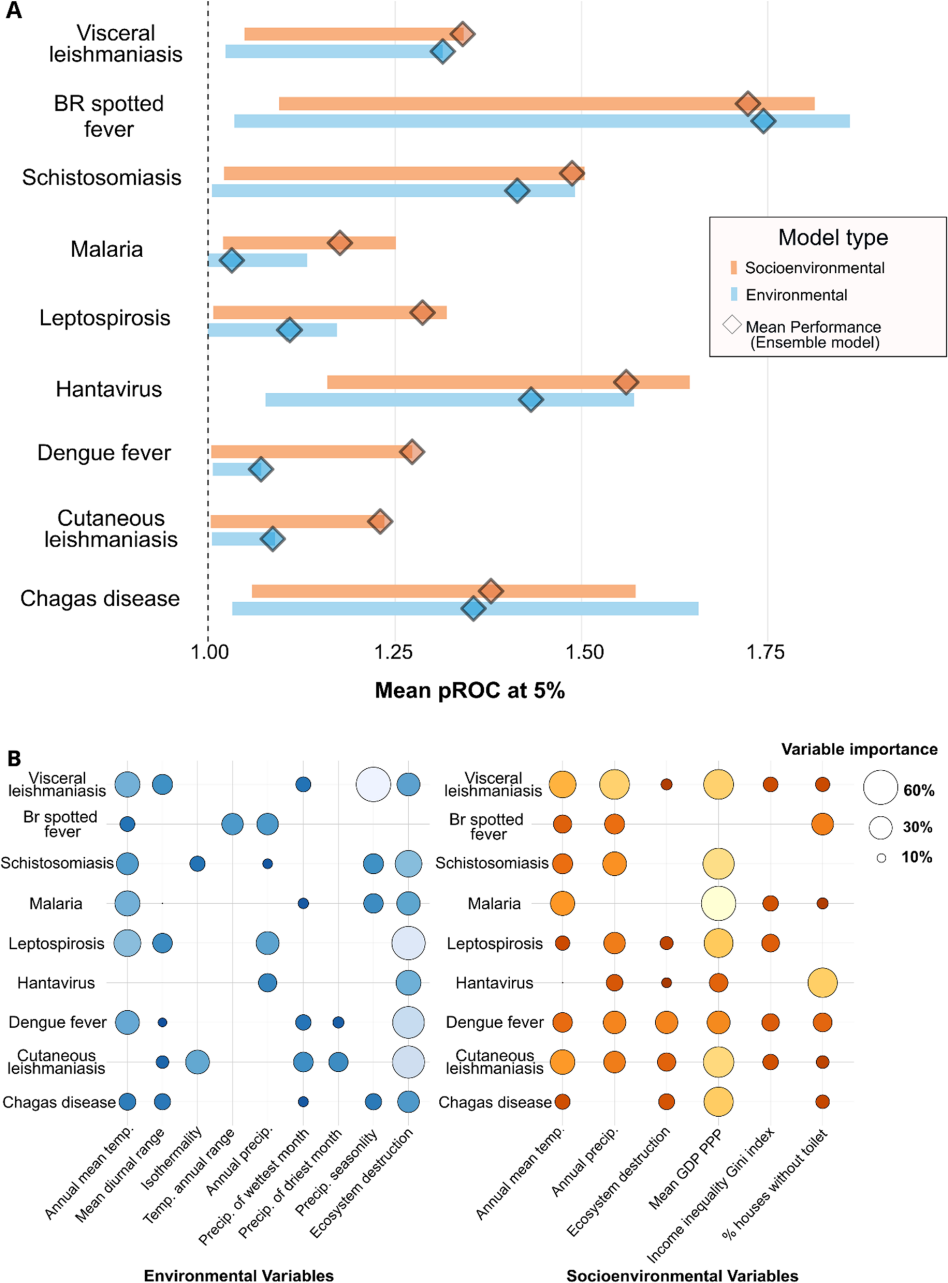
Comparison between models’ performance and each relative variable importance. Overall performance and relative variable importance between ecological niche models and environmental (E) and socioenvironmental (SE) model predictors. **A** Small circles correspond to mean pROC values, generated based on a 5% error threshold, derived by multiple models using different algorithms (Random Forest, Maxent, SVM, and GLM). The diamond shapes represent the values from the ensemble (averaged) models for each disease. Blue represents models made with environmental sets of predictors and orange colors represents models made with socioenvironmental sets predictors. The dashed line corresponds to neutral performance. **B** The relative variable importance of each predictor is represented by circle size, in orange the models were made with environmental set predictors, in blue with socioenvironmental sets of predictors. The relative variable importance varies from 10 to 60%. pROC: Partial receiver operating characteristic; Br spotted fever: Brazilian spotted fever; temp.: temperature; precip.: Precipitation; Mean GDP PPP: Mean gross domestic product using purchasing power parity rates from [43]; % of households without toilet: Percentage of households without toilet from [38]

After incorporating socioeconomic variables into the modeling effort, disease risk areas became more defined (Figs. 2 and 3), particularly for dengue fever, malaria, and cutaneous leishmaniasis, which shifted from mild suitability values covering the entire country to more delimited risk in southeast, central, and northeast Brazil, as well as along the Amazon River basin(Figs. 2 and 3). Model performance for dengue fever, malaria, cutaneous leishmaniasis, and leptospirosis, improved the most after adding socioeconomic variables (Table 1, Fig. 4A). The addition of socioeconomic variables had no significant effect on the models of Brazilian spotted fever, visceral leishmaniasis, and Chagas disease (Table 1, Fig. 4A), and actually slightly decreased the performance of the spotted fever model (change in pROC = −1.1%).

### Poverty and disease risk

**Fig. 5:**
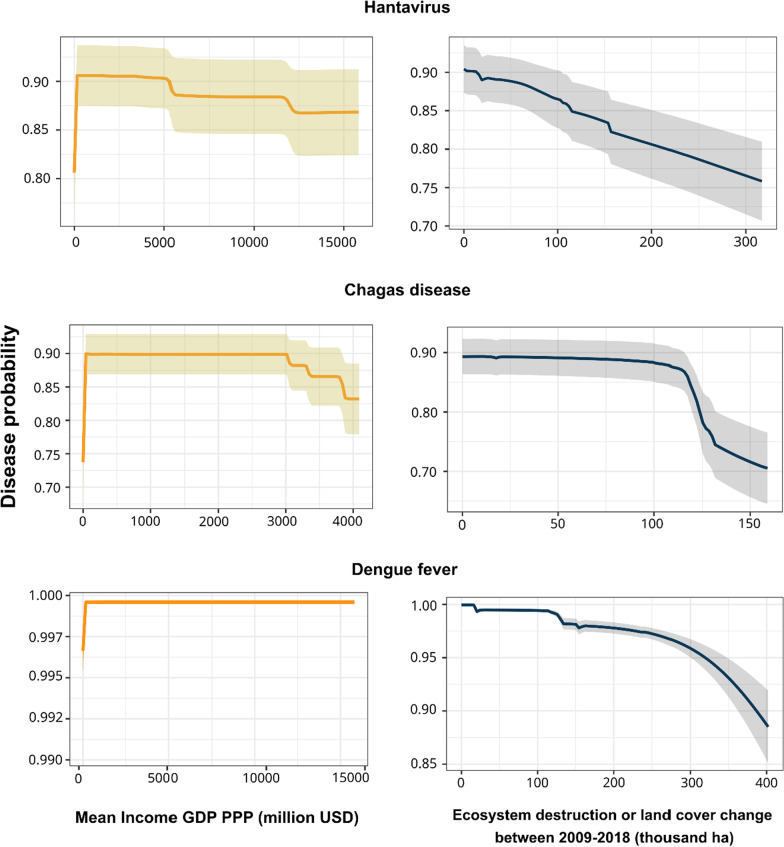
Model individual response curves related to environmental (blue and gray) and social (orange and dark yellow) variable gradients, calculated using evaluating strips method. Dengue fever, hantavirus and Chagas disease results are shown as they illustrate different responses of disease risk to income and ecosystem destruction The Y-axis corresponds to the probability predicted by the models for disease presence while the X-axis has the predicted values of response variables. The relationship between remaining variables and disease probability, as well as other diseases and environmental and social predictors, is available in Additional file 1: Fig. S1–S9. Mean GDP PPP: Mean gross domestic product using purchasing power parity rates from [43]; ha: hectare

In the composite models, The most significant predictive variables for all diseases were GDP (relative variable importance = 37 ± 13% standard error), followed by annual amount of precipitation (29 ± 7%) and annual mean temperature (24 ± 8%). Low GDP (at or below the poverty level) was associated with higher probability of disease presence, with the strongest effect being found for hantavirus and Chagas disease (Fig. 5). A low GDP was linked to a higher likelihood of sickness, with the exception of dengue fever, which had a positive correlation with GDP (Figs. 4B, 5, Additional file 1: Fig. S3). GDP was the most important socioeconomic variable in the combined models for seven of the nine studied illnesses: schistosomiasis, leptospirosis, malaria, dengue fever, visceral and cutaneous leishmaniasis, and Chagas disease. For hantavirus and Brazilian spotted fever, theproportion of households without toilets was the most significant disease risk predictor (42% and 27% of relative variable importance respectively; Fig. 4B).

### Ecosystem destruction and disease risk

In the simple models, the most important environmental predictor for most diseases (except for Brazilian spotted fever) was ecosystem destruction between the years of 2008 and 2019 (mean relative variable importance of 42%), followed by annual mean temperature (32%, Fig. 4). Ecosystem destruction was the most important variable of environmental models for six of the nine studied diseases: leptospirosis, cutaneous leishmaniasis, dengue fever, schistosomiasis, hantavirus, and Chagas disease. Overall, ecosystem destruction had 31% more importance in determining disease presence than the next variable which was mean annual temperature. The relationship between ecosystem destruction and disease likelihood exhibited a negative trend, which meant ecosystem destruction increased disease likelihood when its levels were low (Fig. 5).

## Discussion

Understanding the environmental and socioeconomic predictors which determine disease probability and vulnerability in human populations is a core question in disease surveillance and prevention. Untangling the effects of these variables can help predict transmission events and spatiotemporal emergence of outbreaks, which allow us to forecast the origins and paths of epidemic spread. Here, we show how ecosystem destruction and poverty correlates with the presence of several life cycle zoonoses and neglected tropical diseases on large geographic areas (Fig. 3). Disease probability was relatively high when GDP and natural ecosystem destruction were low, corresponding to its early stages. Furthermore, adding socioeconomic variables in disease risk modeling improved overall model performance and accuracy by up to 18%. Likewise, socioeconomic variables significantly contribute to habitat suitability modeling and disease risk mapping for several neglected tropical and zoonotic diseases.

Disease modeling approaches supported by ecological niche theory have additional advantages and applications concerning traditional spatial epidemiology models, including a biological understanding of the drivers of disease occurrence [22]. Climate-only ecological niche models, however, may end up identifying areas with the environmental conditions suitable for the pathogen, vector, or reservoir, without accounting for the presence of vulnerable human populations or densely populated cities. We found that dengue fever, malaria, Chagas disease, and hantavirus models had a more delimited distribution when susceptible populations were considered as a force facilitating the long-term maintenance of transmission (Fig. 2). Our findings also demonstrate that the absence of socioeconomic variables in disease transmission models may result in a misleading disease transmission risk, which in turn can have a negative influence on public health policy aiming to allocate resources for disease prevention and treatment [1]. More precision is a desirable modeling feature when aiming to conduct data-driven surveillance or deployment of vaccines or treatment to at-risk populations outside the traditional disease-control plans.

Almost all diseases had GDP as a crucial socioeconomic variable, with a tendency to have a higher probability of disease at low-income levels, (i.e., high poverty). This pattern was demonstrated more strongly for Chagas disease and hantavirus (Fig. 5). Overall, these results suggests that poorer populations may have higher probability of disease transmission risk. In fact, poverty is usually associated with infectious disease risk and susceptibility [32, 33]. Low GDP has been linked to malaria, dengue, and a variety of other infectious diseases, where it may act as both a cause and a consequence of disease risk [31, 42]. This relationship between poverty and neglected tropical diseases may hinder populations and communities from escaping disease risk, a process known as poverty trap [32]. Poverty traps may lock communities into a cycle of diseases and poverty that may last generations if interventions with specific policies are not implemented [58]. As a result, public health policies must be combined with social policies that reduce poverty.

In contrast, GDP was positively associated with dengue fever risk (Figs. 2 and 5), where municipalities with higher income also had higher risk of infection. This could be associated with the capacity of dengue transmission to be better sustained is urban areas with high population densities [3]. An analogous result was obtained with Brazilian spotted fever, which had a negative relationship with the proportion of households without a toilet (Additional file 1, Fig. S6). One explanation for this pattern could be that cities with high proportion of houses without toilet are in the Amazon region where Brazilian spotted fever is less prevalent [38]. Brazilian spotted fever occurs in areas with more intense agriculture and cattle economy, towards the central part of the country. These unexpected results encompassing Brazilian spotted and dengue fever demonstrates how different socioeconomic status proxies can denote complex transmission-risk gradients.

We also observed that disease transmission risk for dengue, cutaneous leishmaniasis, Chagas disease, schistosomiasis, leptospirosis, and hantavirus increases under moderate to low levels of ecosystem degradation, in agreement with similar findings on malaria (Fig. 5) [31]. The early stages of ecosystem degradation could alter parasite transmission cycles surrounding pristine forests and savannas facilitating disease emergence [31]. In the case of malaria, the mosquito vector *Anopheles cruzii* is found in great abundance in tree canopies, but during deforestation the mosquito shifts to ground level increasing malaria prevalence in both simians and humans [59]. This driver of disease emergence is also related to spillover events, which occur when a parasite from a natural cycle of transmission infects a different species, in this case humans [60], which has been observed during deforestation events [30]. The link between land-use change and outbreaks has been demonstrated recently by epidemics such as Ebola [61] and COVID-19 [62]. The gradient of recent deforestation or native vegetation loss in Brazil suggests that active deforestation was widespread in municipalities across the country [40] (see correlation of variables at Additional file 2). Thus, municipalities that went through ecosystem degradation in the past, but do not show ongoing landscape change, may demonstrate different transmission patterns and lower disease risk. Active deforestation and landscape fragmentation may lead to peaks in disease transmission. Our study shows insights on how disease transmission risk varies along ecosystem destruction gradients and how the age of landscape destruction can be used to model vulnerability to disease across large-scale study areas like Brazil.

Our findings also support the argument that disease-transmission risk is driven by the confluence of poverty and ecosystem destruction. Thus, human population health security cannot be achieved without addressing associated sectors such as ecosystem health. Our results indicate that it may be necessary to halt deforestation, even in its early stages to prevent spillover events and new outbreaks. This concept is specifically highlighted in the One Health approach [5] and it is coherent with the United Nations Sustainable Development Goals and the Global Health Security Agenda [6]. Additionally, our results highlight the importance of multiple sustainable development goals and provide an example of how they are interrelated: to promote, prevent, and surveil good health and well-being it is necessary to extinguish poverty in its different forms and to ensure the protection of terrestrial ecosystems. These are central themes attuned by United Nations by the first, third, and fifteenth sustainable development goals. Unfortunately, actions towards poverty eradication, health and well-being promotion, and the protection of terrestrial land had been reduced by the SARS-CoV-2 ongoing pandemic, which made these themes even more urgent. Thus, to prevent neglected tropical diseases that have strong relationship to poverty and environment destruction, is necessary encompassing common solutions among the divergent development goals.

It is worth noting that our analyses could have potential limitations. As aforementioned, potential diagnostic bias, particularly in terms of regional variations in the capacity to detect diseases, can produce low specificity and sensibility in the health surveillance system, especially in the Amazon region [13]. However, the good reach capacity of Brazilian healthcare [63] and our presence-only approach, which is more conservative, can mitigate this problem. Furthermore, we used 2010 socioeconomic parameters, and it is possible that supplying outdated variables to the model will affect its accuracy and applicability or bias the models. However, we observed that socioeconomic variables in Brazil, at a large scale, are highly correlated and retain their spatial patterns regardless of timeframe. While the current Brazilian government has been slow to release socioeconomic census data [64], we believe that it was the best decision to use the variables from the 2010 socioeconomic census to not further hamper the importance of this disease surveillance effort. Thus, we believe our analysis and interpretation of the results are sound. These and other limitations are discussed in greater detail in Additional file 1.

Public health strategies in Brazil should be directed to economically disadvantageous populations adjacent to areas undergoing fast ecosystem destruction. We found that the high socioeconomic inequalities and disparities widespread in Brazil are expected to exacerbate the burden of neglected tropical diseases. This is particularly remarkable given the recent country’s increasing scenario of severe and rapid deforestation, which is influenced by ongoing deliberate decisions from the Brazilian government [65]. For example, during the administration of president Jair Bolsonaro (2019–2022), Brazil’s environmental agency spent less than half of its budget to protect the environment in 2021 despite the record-breaking deforestation [66]. Also in 2021, Brazil was in a severe economic crisis, making populations in poverty even more vulnerable to disease outbreaks. Strikingly, scientists in Brazil now have limited access to updated data on both the socioeconomic-demographic census and infectious disease occurrences at the national level implying that critical decisions are being conducted with incomplete information. To achieve satisfactory public health results and sustainable development, Brazil must recover disease surveillance and sociodemographic census on a country scale and implement policies aiming to reduce socioeconomic imbalances and destruction of natural habitats.

## Conclusions

Neglected tropical and zoonotic diseases are main explained, at macro-scales by poverty and early ecosystem destruction in Brazil. To disrupt the cycle of disease transmission, it is vital to ensure that strategies for public health are aligned with policies of poverty diminishment and ecosystem conservation policies. Seeking equitable public health interventions and socioeconomic rescue well aide in disrupting poverty cycles, balanced with coordinated country-specific strategies on deforestation suppression, should be a continental priority to control the neglected tropical diseases and prevent poverty traps caused by a combination of environmental and social factors that facilitate disease emergence and transmission.

## Supplementary Information

**Additional file 1.** This material comprises several components, including an assessment of potential limitations, response curves for all diseases analyzed, both in composite and simple models, an exhaustive list of the variables employed in the analysis, and a comprehensive model reproducibility checklist containing essential details about disease occurrence and data processing.

**Additional file 2.** This file includes a comprehensive tutorial on the construction of niche models and maps, utilizing the R programming language with instructive guidance.

## Data Availability

The data and code to reproduce the analyses are available in the Additional files1, 2 and in in the GitHub repository: https://github.com/arthurama/poverty-and-habitat-loss-are-predictors-of-NTD-in-Brazil.git.

## Abbreviations

Datasus: Informatics Department of the Unified Brazilian Health System
IBGE: Brazilian institute of geography and statistics
GDP: Gross domestic product
pROC: Partial receiver operating characteristic
AUC: Area under the curve
