# Supplementary material for: Neglected tropical diseases risk correlates with poverty and early ecosystem destruction
Source: Infect Dis Poverty. 2023 Apr 10;12:32. doi: 10.1186/s40249-023-01084-1 (PMC10084676; doi:10.1186/s40249-023-01084-1)
Supplement: Supplementary file 1 — Additional file 1. This material comprises several components, including an assessment of potential limitations, response curves for all diseases analyzed, both in composite and simple models, an exhaustive list of the variables employed in the analysis, and a comprehensive model reproducibility checklist containing essential details about disease occurrence and data processing. [file 40249_2023_1084_MOESM1_ESM.docx]

**Additional files**

**Neglected tropical diseases risk correlates with poverty and early ecosystem destruction**

**Authors:** Arthur Ramalho Magalhães^1^, Cláudia Torres Codeço^2^, Jens-Christian Svenning^3^, Luis Escobar^4,5^, Paige Van de Vuurst^5,6^ and Thiago Gonçalves-Souza^1^*

**Affiliations**

^1^ Laboratory of Ecological Synthesis and Biodiversity Conservation (ECOFUN), Federal Rural University of Pernambuco, Recife, PE, Brazil

^2^ Scientific Computation Program (PROCC), Oswaldo Cruz Foundation (Fiocruz), Rio de Janeiro, RJ, Brazil

^3^ Center for Biodiversity Dynamics in a Changing World (BIOCHANGE) & Section for Ecoinformatics & Biodiversity, Department of Biology, Aarhus University, Aarhus, Denmark

^4^ Department of Fish and Wildlife Conservation, Virginia Tech, Blacksburg, VA, USA

^5^ Center for Emerging Zoonotic and Arthropod-borne Pathogens, Virginia Tech, Blacksburg, VA, USA

^6^ Translational Biology, Medicine and Health Program, Virginia Tech Graduate School, Blacksburg, VA, USA

**Corresponding author Email:** tgoncalves.souza@gmail.com

**Table Of Contents**

Supplementary text

Figures S1 to S11

Tables S1 to S2

Legends for Datasets S1

SI References

**Other supplementary materials for this manuscript include the following:**

Rmarkdown (R-code for reproducibility)

Dataset for analysis reproducibility is available at:

https://github.com/arthurama/poverty-and-habitat-loss-are-predictors-of-NTD-in-Brazil.git

Text

**Potential limitations**

One of the main constraints of our approach is that disease occurrence was connected to cities' official coordinates, which were often the cities’ administrative center. Accurate addresses are unavailable due to the private nature of the information. Furthermore, this association to the cities’ administrative center distorts climatic and environmental variables, especially given the size of some municipalities. Towards west side and in the Amazon regions municipalities are larger, and by extension this spatial uncertainty could increase the augmentation of our background data and disease occurrences. Furthermore, the models mostly ignored the socioeconomic diversity seen in Brazilian municipalities [1]. As a result, larger cities are prone to higher mean GDP, HDI, and hence had a higher possibility of recording a disease presence. In this way, high GDP and HDI could be associated with higher probability of occurrence of some diseases. In other words, the effect of these socioeconomic variables could be redirected as an undesired outcome. We used the inequality index to mitigate this problem (Gini coefficient [1]). We hope that future works could have district level specific data, being able to insert inner cities’ variability that could lay more layers of information to overall disease patterns.

Additionally, that are some particularities about the Brazilian public health diagnostic capacity that should be taken into consideration. For instance, regional discrepancies in surveillance coverage are very strong in Brazil, with some regions receiving more resources than others thus affecting diagnosis and lab testing [2,3]. Although this could impact the risk delimitation of the diseases in question, we believe that since we used a disease presence-only per municipality approach and a relatively large window of time, those discrepancies were alleviated. Presence-only models are more conservative and recent studies evaluating species distribution models have regarded the presence-only models as the most successful in predicting independent data. Moreover, 2010 socioeconomic parameters such as Gini index and percentage of households without toilet per municipalities were used as inputs, so it's possible that using out-of-date variables affect how accurate our models are. However, we found that Brazil's socioeconomic variables are highly correlated and maintain their spatial patterns. For instance, we tested the correlation between Mean GDP per capta [4] in Brazil between the years 1990 and 2016 and obtained 96% correlation. While the current Brazilian government is slow to release data for analysis, we believe that it was the best decision to use the variables from the 2010 socioeconomic census to not further hamper the importance of this disease surveillance effort. It is also worth of noting that adding socioeconomic variables did not improve models for all diseases, which was the case of tick-borne Brazilian spotted fever (Table 1). Whether these results are fully or in part due to data limitations or the specific natural history of the diseases is unknown. Alternatively, sampling bias could be considered a driver of overall model improvement during the inclusion of socioeconomic variables by improving model fit in areas with more sampling effort due to more resources, more people, or better surveillance [5]. We mitigated this problem by measuring model performance by splitting the data to generate independent datasets for model evaluation and by measuring success in the prediction of new cases instead of measuring model fit (see Methods). Additionally, we used presence-only models in large windows of time and did not assess inter-year variation in occurrences that could be strongly correlated to seasonality.

Furthermore, the black box approach used [6] disregards disease cycle particularities such as the distribution (or occurrence) of vectors involved, the distribution of hosts, and taxonomic differences between parasites of the same diseases or virus serotypes, which may have differences in disease niches such as preferring different hosts [7]. Although these differences between disease particularities do not fit the scope of this article, as we did not aim at investigating intra-disease potential differences in socioenvironmental niches, we would like to recognize that they may have had an impact on the modeled niches constructed in this work.


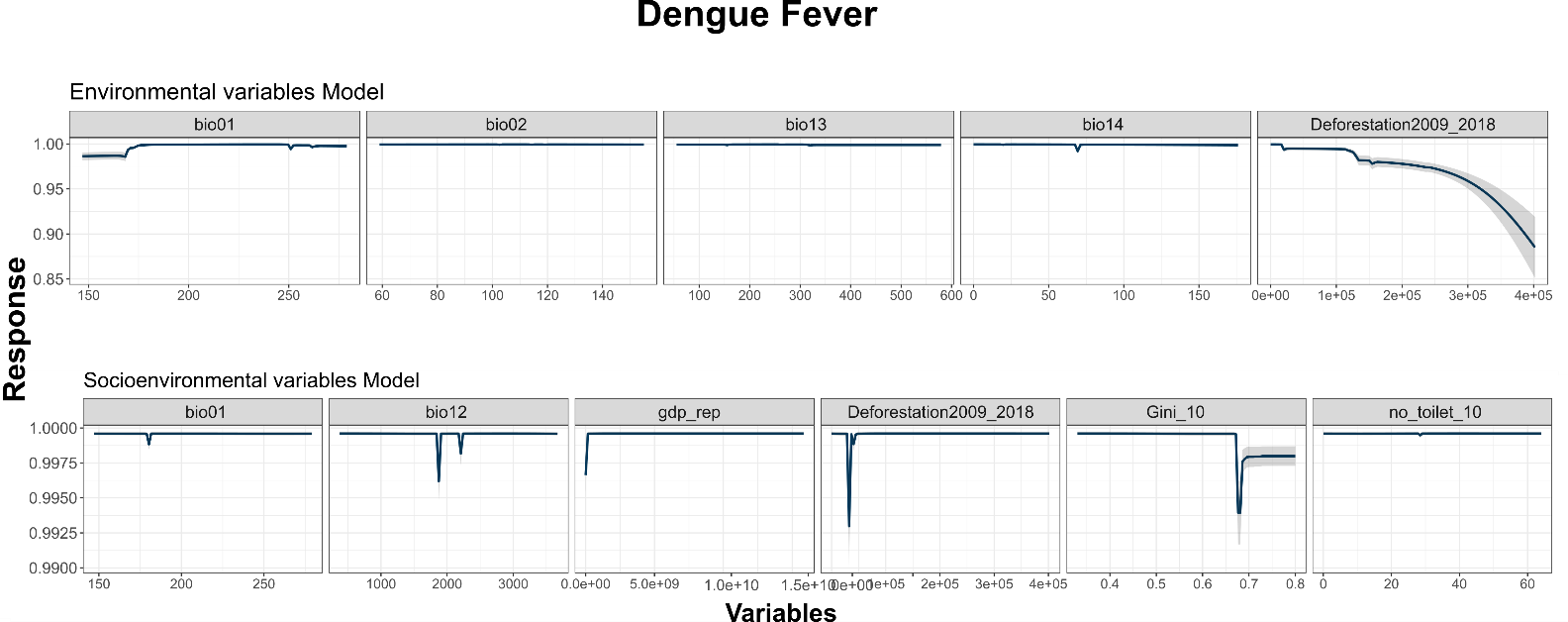
Fig. S1. Response curves of each predictor variable used in the environmental variables model (simple model) and socioenvironmental variable model (composite model) of Dengue Fever. The curves represent how the probability of Dengue fever presence reacts to the variable specified gradient. The response curves are generated as mean response curves of 60 fitted models among different algorithms: random forest, glm, svm, and maxent. The gray area represents 95% of confidence interval.


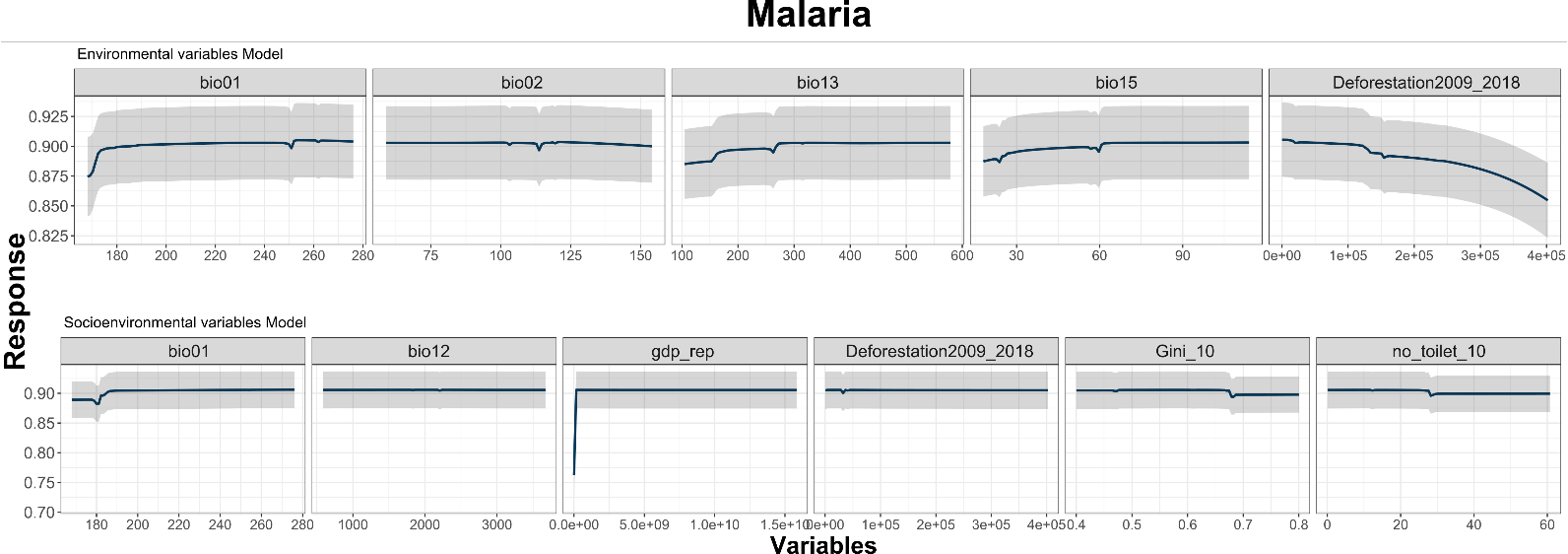


Fig. S2. Response curves of each predictor variable used in the environmental variables model (simple model) and socioenvironmental variable model (composite model) Malaria. The curves represent how the probability of Malaria presence reacts to the variable gradient. The response curves are generated as mean response curves of 60 fitted models among different algorithms: random forest, glm, svm, and maxent. The gray area represents 95% of confidence interval.


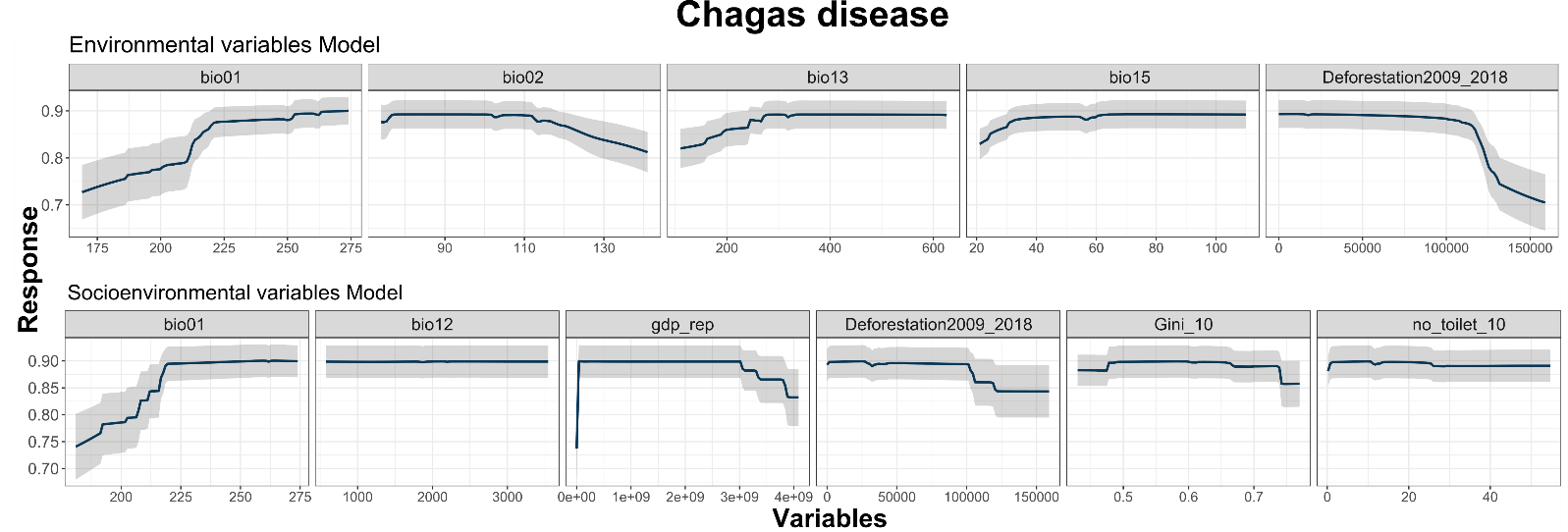


Fig. S3. Response curves of each predictor variable used in the environmental variables model (simple model) and socioenvironmental variable model (composite model) of Chagas disease. The curves represent how the probability of Chagas disease presence reacts to the variable gradient. The response curves are generated as mean response curves of 60 fitted models among different algorithms: random forest, glm, svm, and maxent. The gray area represents 95% of confidence interval.


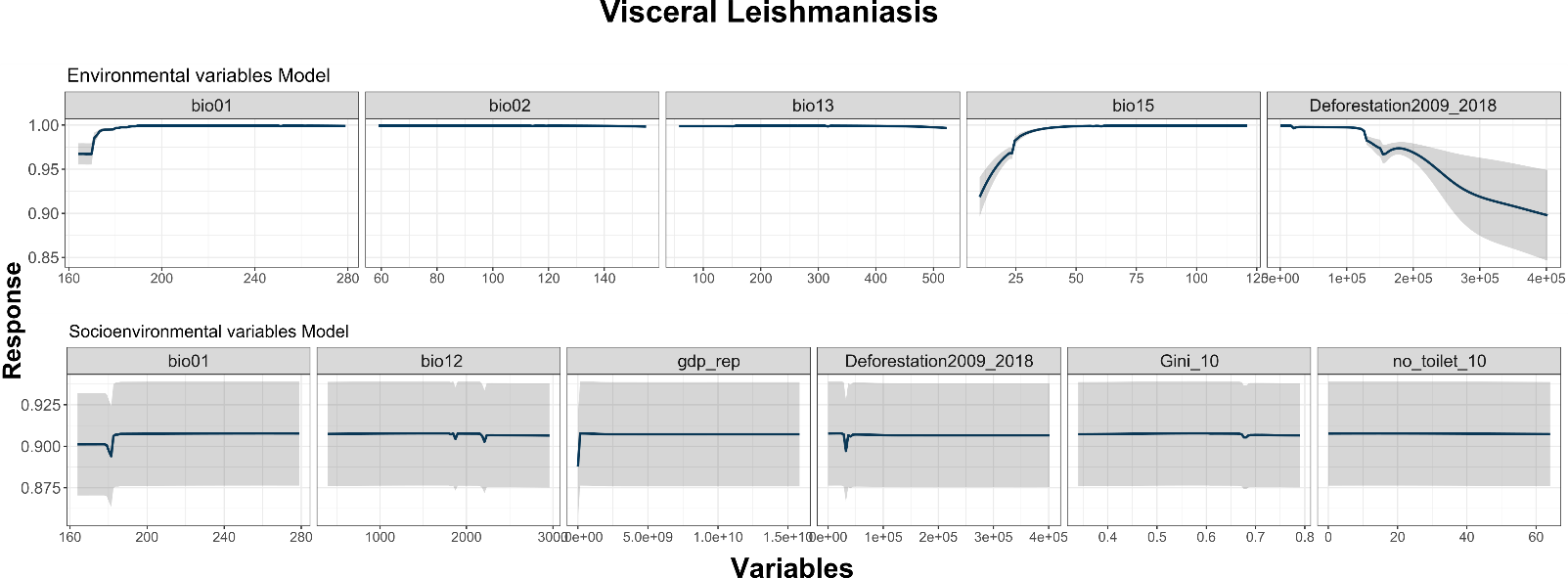


Fig. S4. Response curves of each predictor variable used in the environmental variables model (simple model) and socioenvironmental variable model (composite model) of visceral leishmaniasis. The curves represent how the probability of visceral leishmaniasis presence reacts to the variable gradient. The response curves are generated as mean response curves of 60 fitted models among different algorithms: random forest, glm, svm, and maxent. The gray area represents 95% of confidence interval.


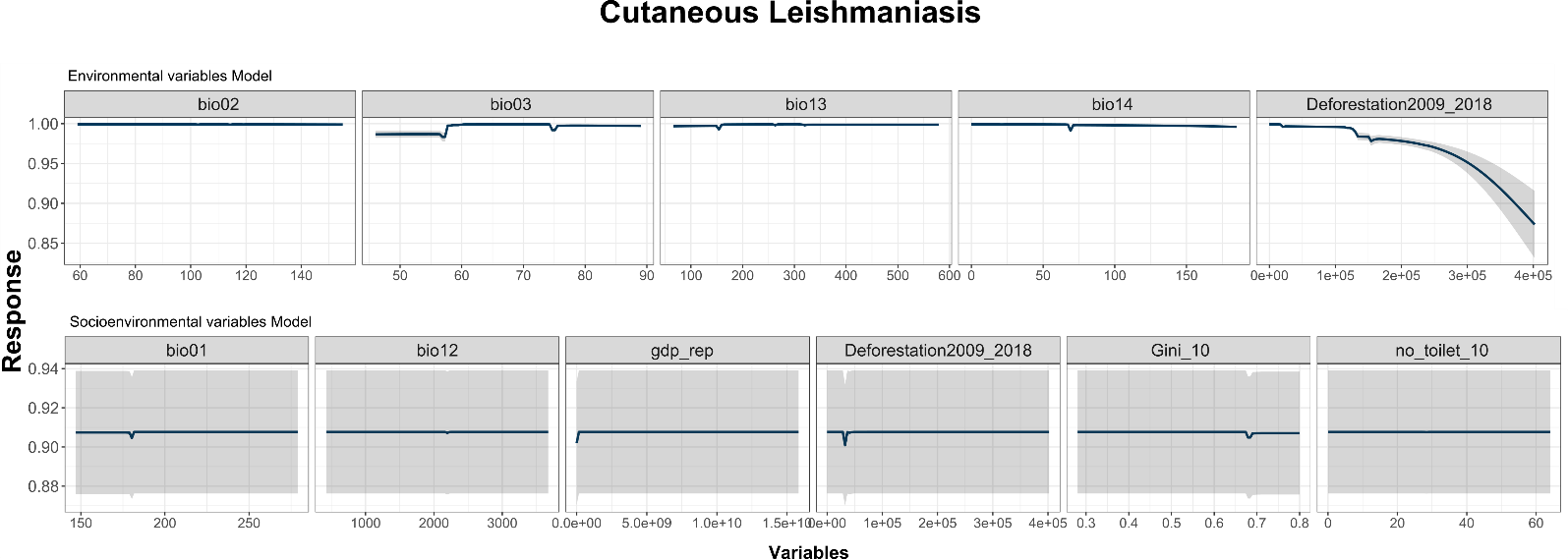


Fig. S5. Response curves of each predictor variable used in the environmental variables model (simple model) and socioenvironmental variable model (composite model) of cutaneous leishmaniasis. The curves represent how the probability of cutaneous leishmaniasis presence reacts to the variable gradient. The response curves are generated as mean response curves of 60 fitted models among different algorithms: random forest, glm, svm, and maxent. The gray area represents 95% of confidence interval.


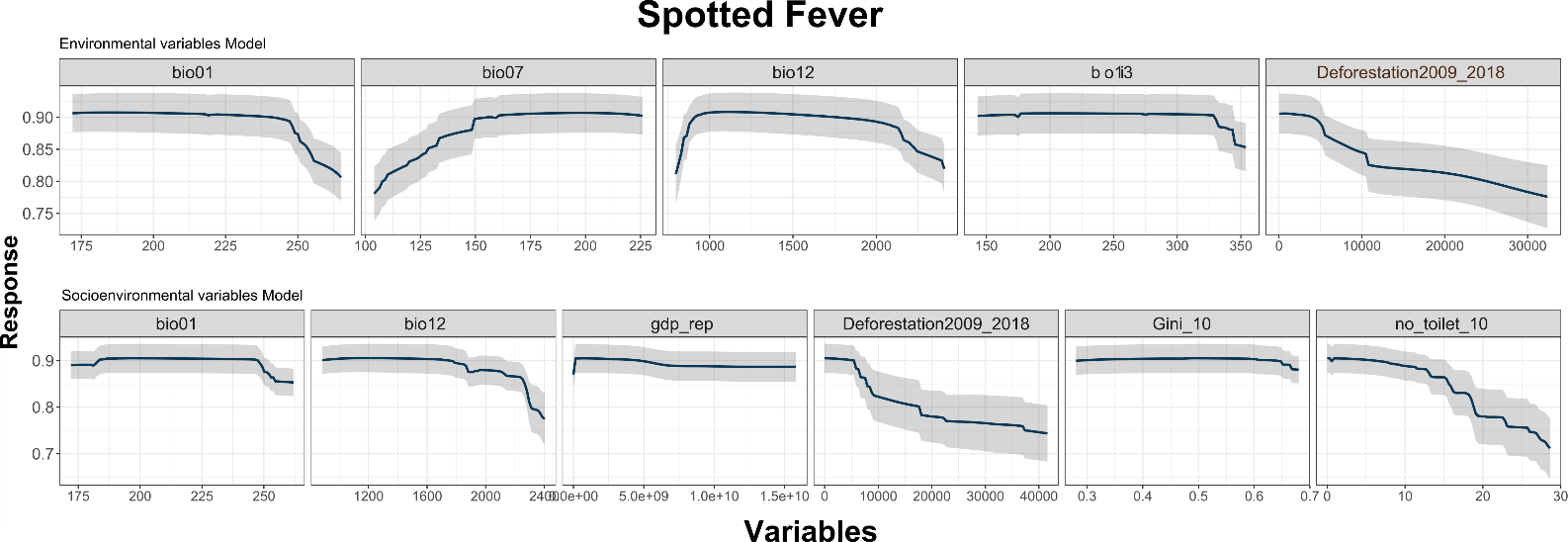


Fig. S6. Response curves of each predictor variable used in the environmental variables model (simple model) and socioenvironmental variable model (composite model) of spotted fever. The curves represent how the probability of spotted fever presence reacts to the variable gradient. The response curves are generated as mean response curves of 60 fitted models among different algorithms: random forest, glm, svm, and maxent. The gray area represents 95% of confidence interval.


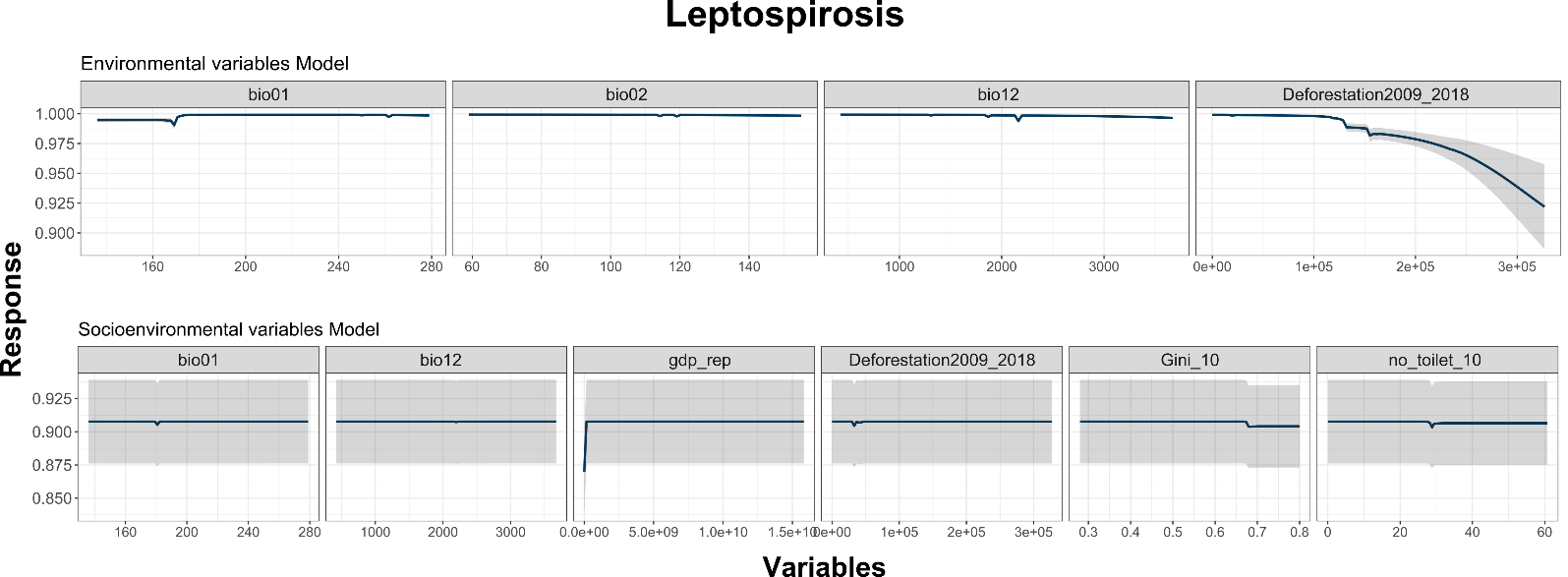


Fig. S7. Response curves of each predictor variable used in the environmental variables model (simple model) and socioenvironmental variable model (composite model) of leptospirosis. The curves represent how the probability of leptospirosis presence reacts to the variable gradient. The response curves are generated as mean response curves of 60 fitted models among different algorithms: random forest, glm, svm, and maxent. The gray area represents 95% of confidence interval.


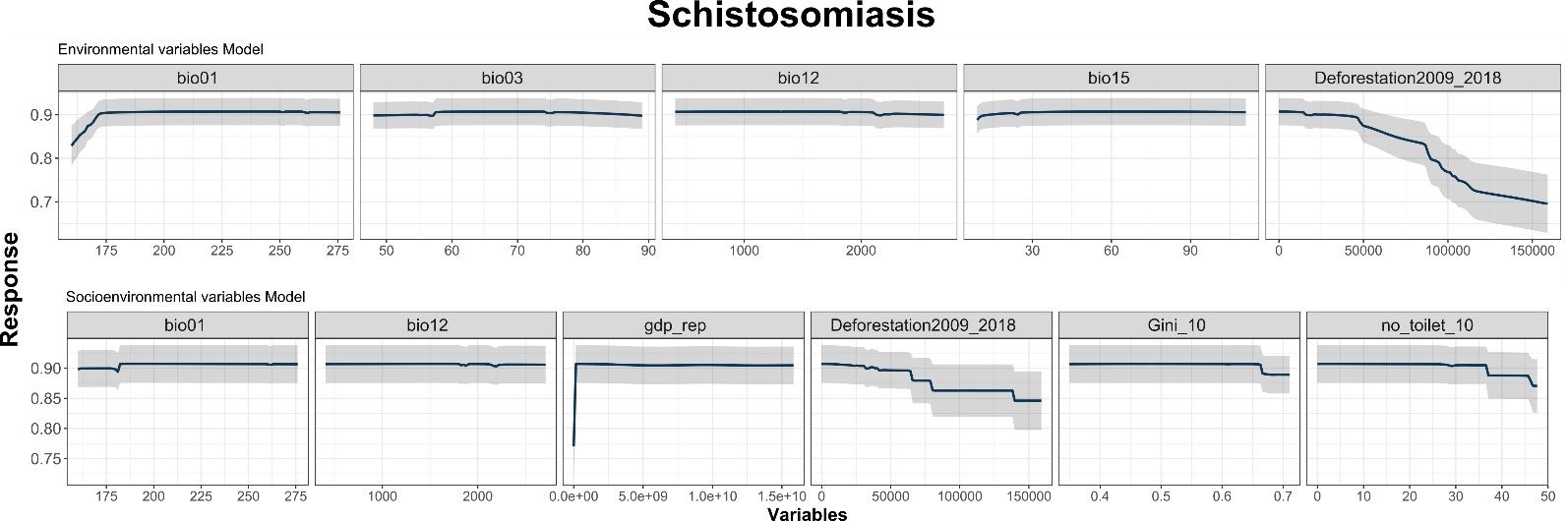


Fig.S8. Response curves of each predictor variable used in the environmental variables model (simple model) and socioenvironmental variable model (composite model) of schistosomiasis. The curves represent how the probability of schistosomiasis presence reacts to the variable gradient. The response curves are generated as mean response curves of 60 fitted models among different algorithms: random forest, glm, svm, and maxent. The gray area represents 95% of confidence interval.


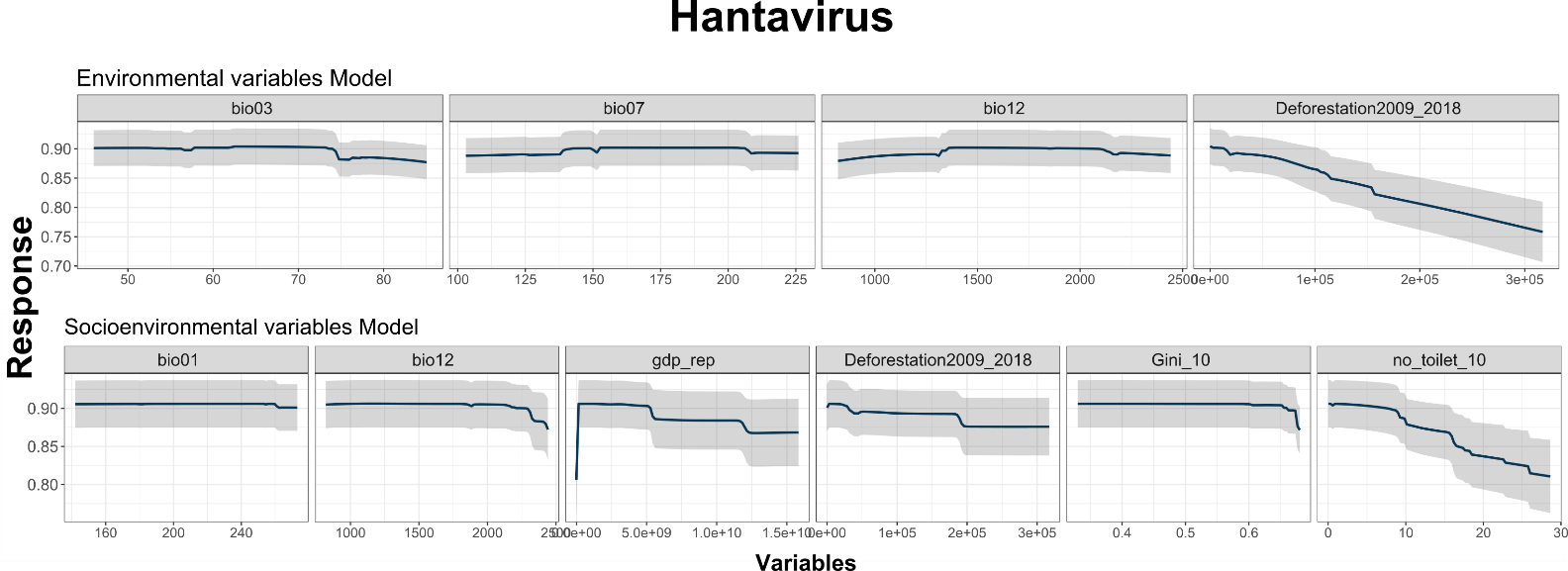


Fig. S9. Response curves of each predictor variable used in the environmental variables model (simple model) and socioenvironmental variable model (composite model) of hantavirus. The curves represent how the probability of hantavirus presence reacts to the variable gradient. The response curves are generated as mean response curves of 60 fitted models among different algorithms: random forest, glm, svm, and maxent. The gray area represents 95% of confidence interval.

Table S1. Model reproducibility checklist based on Feng et al. (2019). Here are described details about disease occurrence data and data processing.

| **Model reproducibility checklist**  **(based on** [11]**)** | **Category** | **What to report** |  | **Rationale** |
| --- | --- | --- | --- | --- |
| **Obtaining and processing occurrence data** | **Metadata** | **Source of occurrence data** | Brazilian ministry of health | - |
|  |  | **download date/version data source** | fev/21 | - |
|  |  | **Basis of records** | DATASUS- SINAN - link: https://datasus.saude.gov.br/acesso-a-informacao/doencas-e-agravos-de-notificacao-de-2007-em-diante-sinan/ | - |
|  |  | **Spatial extent** | Brazil political limits | - |
|  |  | **All diseases Temporal range** | 2007-2018 |  |
|  |  | Dengue fever | 2013-2016 | Cases beyond 2016 and before 2013 had no explicit laboratory confirmation |
|  |  | Malaria | 2011-2017 | Before 2011 cases do not exhibited laboratory confirmation on the database |
|  |  | Chagas disease | 2007-2018 | Cases beyond 2018 were not available at the database |

Table S1. (Continued)

| **Model reproducibility checklist (based on Feng et al.2019)** | **Category** | **What to report** |  | **Rationale** |
| --- | --- | --- | --- | --- |
|  |  | Visceral Leishmaniasis | 2007-2018 | Cases beyond 2018 were not available at the database |
|  |  | Cutaneous leishmaniasis | 2007-2018 | Cases beyond 2018 were not available at the database |
|  |  | Spotted fever | 2007-2013 | Cases beyond 2013 were not available at the database |
|  |  | Schistosomiasis | 2008-2017 | Cases beyond 2017 were not available at the database |
|  |  | Leptospirosis | 2007-2017 | Cases beyond 2017 were not available at the database |
|  |  | Hantavirus | 2007-2014 | Cases beyond 2014 were not available at the database |
|  | **Processing** | **Duplicate coordinates** | Removed. Only one occurrence (presence) was left by municipality, therefore filtering duplicates out. |  |
|  |  | **Spatial environmental outlier error** | Not treated. |  |
|  |  | **Spatial coordinate uncertainty** | Not treated. All occurrences were assigned to municipalities' official coordinates since the precise address of patients are occulted as sensitive data. |  |
|  |  | **Sampling Bias** | Municipalities sizes and disposition of resources in hospitals over Brasil. |  |
|  |  | **Spatial autocorrelation** | Treated. using a 18 km side grid. Only one occurrence was left by cell. |  |

Table S2. List of all variables originally proposed to the analysis. Only few variables were left after autocorrelation assessment and entered the final analysis and construction of socioenvironmental variable model (composite model) and environmental only variable model (simple model). All variables had their original resolution changed to 10 minutes to match the worldclim bioclimatic variables.

| **Variables** | **Code** | **Unit** | **Meaning** | **Original Spatial Resolution** | **Entered the final model** | **Time Range** | **Download date** | **Source** | **Reference** | **Access link** |
| --- | --- | --- | --- | --- | --- | --- | --- | --- | --- | --- |
| ***Climatic*** | | | | | | | | | | |
| **Temperature derived** | bio 01-bio 03 | ºC x 100 | Temperature-derived climatic variables | 10' | only bio 01 | averaged for the period 1970-2000 | 28/05/2020 | Worldclim 2 | [8] | <http://www.worldclim.com/version2> |
| **Precipitation derived** | bio 12-bio 15 | mm | Precipitation-derived climatic variables | 10' | only bio 12 | averaged for the period 1970-2000 | 28/05/2020 | Worldclim 2 | [8] | <http://www.worldclim.com/version2> |
| ***Landscape*** | | | | | | | | | | |
| **Habitat loss** | deforestation2009_2018 | hectare | Sum of the area from municipalities which native forest and native grassland that was substituted by pasture, agriculture or urban infrastrucuture | Municipality level | yes | sum of values between 2009-2018 | 15/04/2021 | Mapbiomas v 5.0 | [9] | <https://mapbiomas.org/estatisticas> |

Table S1. (Continued)

| **Variables** | **Code** | **Unit** | **Meaning** | **Original Spatial Resolution** | **Entered the final model** | **Time Range** | **Download date** | **Source** | **Ref.** | **Access link** |
| --- | --- | --- | --- | --- | --- | --- | --- | --- | --- | --- |
| **Native vegetation cover** | native_form_16 | hectare | Municipality native vegetation cover. | Municipality level | no | 2016 | 15/04/2021 | Mapbiomas v 5.0 | [9] | <https://mapbiomas.org/estatisticas> |
| ***Socioeconomic*** | | | | | | | | | | |
| **GDP per cata (PPP)** | gdp | Mean USD multiplied by population in cell | Proxy for mean income | 5-arc minute | yes | 2015 | 01/10/2020 |  | [4] | <https://doi.org/10.1038/sdata.2018.4> |
| **Population density** | pop_density_10 | Inhabitant/km² | Estimated population density per municipality | Municipality level | no | 2010 | 03/06/2020 | CENSO IBGE 2010  Sidrar R package - table 1301 | [1] | Sidrar package tutorial: https://cran.r-project.org/web/packages/sidrar/vignettes/Introduction_to_sidrar.html |
| **Gini index** | Gini_10 | Degree of inequality | Income inequality by municipality; 0 = no inequality  1 = one individual own all income | Municipality level | yes | 2010 | 03/06/20 | CENSO IBGE 2010;  Atlas de Desenvolvimento Humano no Brasil 2013 | [1,10] | <http://www.atlasbrasil.org.br/acervo/biblioteca> |

Table S2. (Continued)

| **Variables** | **Code** | **Unit** | **Meaning** | **Original Spatial Resolution** | **Entered the final model** | **Time Range** | **Download date** | **Source** | **Reference** | **Access link** |
| --- | --- | --- | --- | --- | --- | --- | --- | --- | --- | --- |
| **Mean IDH** | IDHM_10 | Mean | An index of human development averaged by municipality.  A mean of indices  Income, Education and Longevity, with equal weights. | Municipality level | no | 2010 | 03/06/20 | CENSO IBGE 2010;  Atlas de Desenvolvimento Humano no Brasil 2013 | [1,10] | <http://www.atlasbrasil.org.br/acervo/biblioteca> |
| **Proportion of households with pipped water** | canned_water_10 | % of households per municipality | Proxy for government assistance and vulnerability to disease. | Municipality level | no | 2010 | 30/03/2021 | CENSO IBGE 2010  Sidrar R package - table 3218 | [1] | Sidrar package tutorial: https://cran.r-project.org/web/packages/sidrar/vignettes/Introduction_to_sidrar.html |
| **Proportion of households without toilet** | no_toilet_10 | % of households per municipality | Proxy for house quality and vulnerability to disease. | Municipality level | yes | 2010 | 30/03/2021 | CENSO IBGE 2010  Sidrar R package - table 3218 | [1] | Sidrar package tutorial: https://cran.r-project.org/web/packages/sidrar/vignettes/Introduction_to_sidrar.html |

**Appendix References**

1. IBGE. Censo Demográfico [Internet]. 2010. p. 12. Available from: http://www.sidra.ibge.gov.br/bda/.

2. Codeço CT, Dal’Asta AP, Rorato AC, Lana RM, Neves TC, Andreazzi CS, et al. Epidemiology, Biodiversity, and Technological Trajectories in the Brazilian Amazon: From Malaria to COVID-19. Front Public Heal. 2021;9.

3. Castro MC, Massuda A, Almeida G, Menezes-Filho NA, Andrade MV, de Souza Noronha KVM, et al. Brazil’s unified health system: the first 30 years and prospects for the future. Lancet. 2019;394:345–56.

4. Kummu M, Taka M, Guillaume JHA. Gridded global datasets for Gross Domestic Product and Human Development Index over 1990-2015. Sci Data. 2018;5:1–15.

5. Escobar LE, Qiao H, Cabello J, Peterson AT. Ecological niche modeling re-examined: A case study with the Darwin’s fox. Ecol Evol. 2018;8:4757–70.

6. Johnson EE, Escobar LE, Zambrana-Torrelio C. An Ecological Framework for Modeling the Geography of Disease Transmission. Trends Ecol Evol [Internet]. Elsevier Ltd; 2019;34:655–68. Available from: https://doi.org/10.1016/j.tree.2019.03.004

7. Peterson AT. Mapping Disease Transmission Risk: Enriching Models Using Biogeography and Ecology. Johns Hopkins University Press; 2014.

8. Fick SE, Hijmans RJ. WorldClim 2: new 1-km spatial resolution climate surfaces for global land areas. Int J Climatol. 2017;37:4302–15.

9. Souza CM, Shimbo JZ, Rosa MR, Parente LL, Alencar AA, Rudorff BFT, et al. Reconstructing three decades of land use and land cover changes in brazilian biomes with landsat archive and earth engine. Remote Sens. 2020;12.

10. PNUD, Fundação João Pinheiro, IPEA. Atlas do desenvolvimento humano no Brasil - indice de desenvolvimento humano municipal - IDHM - Metodologia [Internet]. Rio de janeiro; 2013. p. 1–25. Available from: http://www.atlasbrasil.org.br/acervo/biblioteca

11. Feng X, Park DS, Walker C, Peterson AT, Merow C, Papeş M. A checklist for maximizing reproducibility of ecological niche models. Nat Ecol Evol [Internet]. Springer US; 2019;3:1382–95. Available from: http://dx.doi.org/10.1038/s41559-019-0972-5
