# Supplementary material for: Neglected tropical diseases risk correlates with poverty and early ecosystem destruction
Source: Infect Dis Poverty. 2023 Apr 10;12:32. doi: 10.1186/s40249-023-01084-1 (PMC10084676; doi:10.1186/s40249-023-01084-1)
Supplement: Supplementary file 2 — Additional file 2. This file includes a comprehensive tutorial on the construction of niche models and maps, utilizing the R programming language with instructive guidance. [file 40249_2023_1084_MOESM2_ESM.pdf]

Neglected tropical diseases risk correlates with poverty and early ecosystem destruction

Arthur R. Magalhães

27/12/2022

```
library(dplyr)
library(colorRamps)
library(mapr)
library(raster)
library(sf)
library(tidyverse)
library(sdm)
library(knitr)
library(readr)
library(usdm)
library(ENMeval)
library(dismo)
library(tidyr)
require(ENMGadgets)
library(reshape2)
library(maptools)
library(corrplot)
library(beepr)
library(ntbox)
library(sqldf)
library(fields)
library(rgdal)
```

0.1 Model of Dengue Fever Niche

Human cases of dengue fever were gathered from the Brazil health ministry database (DATASUS in 2019 from http://www2.datasus.gov.br/). Only cases recorded between 2007 and 2019 were available. However, only cases between 2013-2016 had lab tests confirmation available and entered the model (mainly by serological tests). As municipality was the thinnest precision available, only one case by municipality entered the model.

Importing dengue fever data

```
a<-("C:/Users/bsmah/OneDrive/Área de Trabalho/novas_bases_de_dados_novas_doencas")

setwd(a)
```

```r
dng<-readr::read_csv("occ_dengue_clean_lim_oppc2.csv")

dng$species<-1
sum(dng$species)
```

```
## [1] 3098
```

This represents the number of municipalities where dengue was laboratory test confirmed between 2007 and 2018.

0.2 Predictor variables

Setting all the predictor variables, in order to capture the environmental and socioeconomic signature from occurrences. set the directory where the predictor variables were saved.

Those were all the variables that originally entered the models: First the Worldclim variables (without the “quarter” variables) and:

```r
plot(var[[1:11]])
```

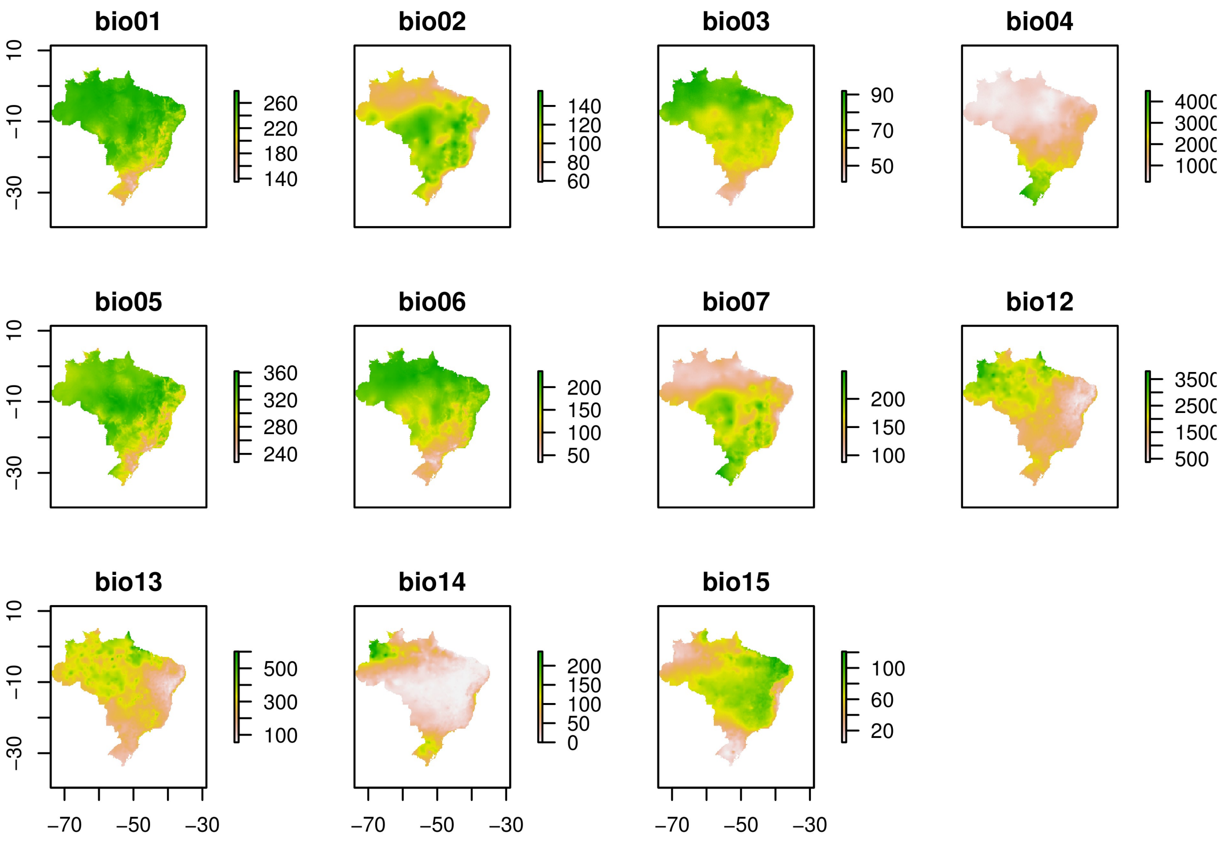


```r
plot(var[[c(12,13,14,16,17,18,19)]])
```

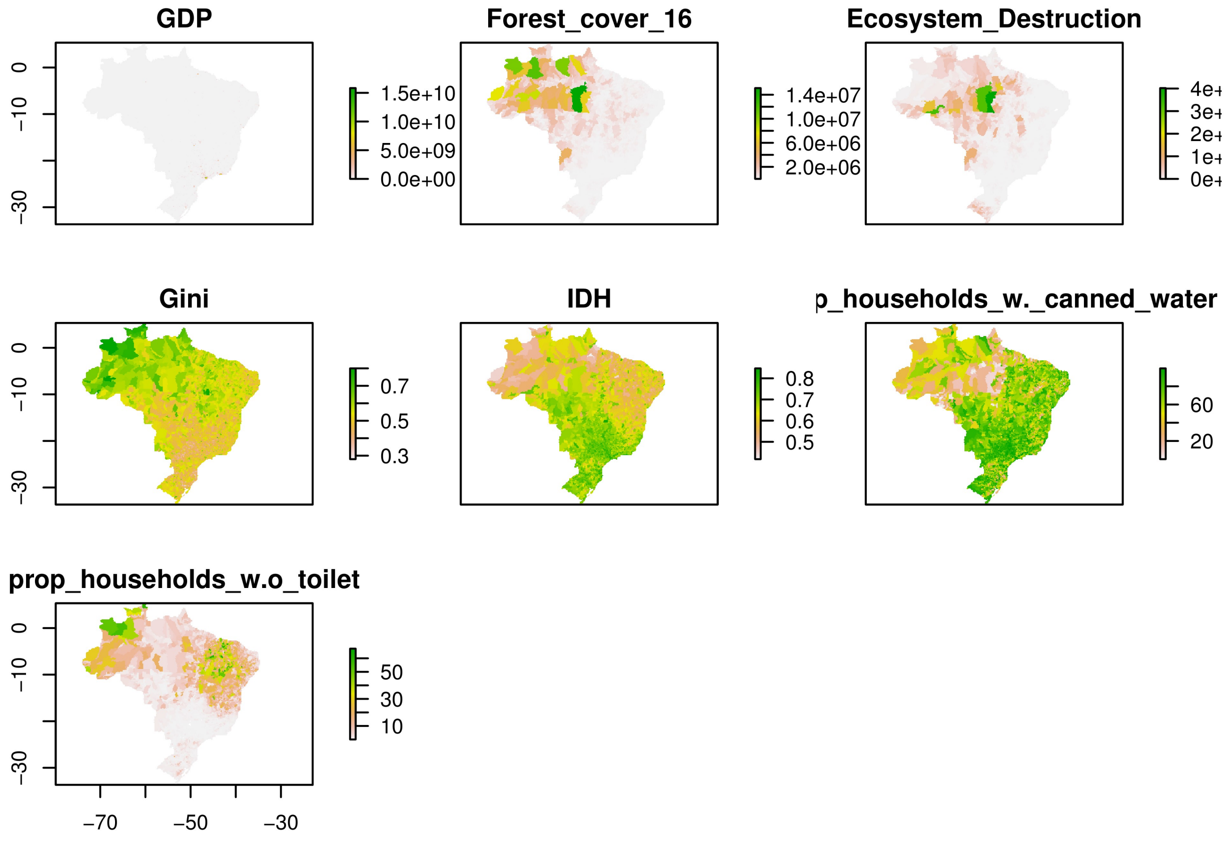

Now, a quick look at the occurrence points inside the variables limits:

```
plot(var[[1]], col='gray')+points(dng2)
```

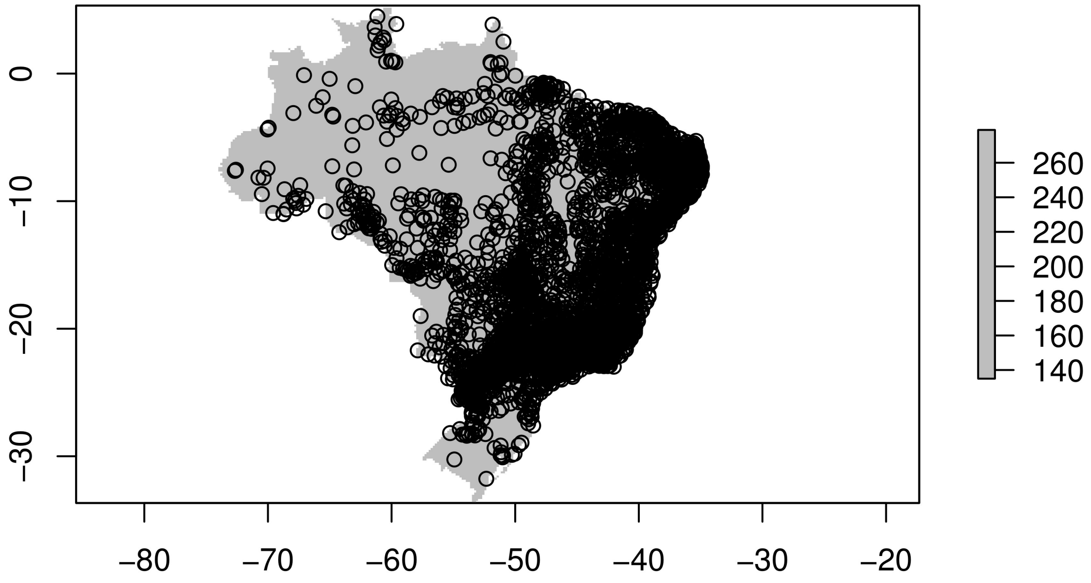


```
## integer(0)
```

0.3 Colinearity and most representative variables

Before choosing variables, we should have a quick check in the variables colinearity to see how they correlate to each other.

```
var.da <- var %>%
  raster::values() %>%
  na.omit

corr <- cor(var.da, method = "spearman")
corrplot:: corrplot.mixed(corr, tl.srt = 45, mar = c(3, 0.5, 2, 1))
```

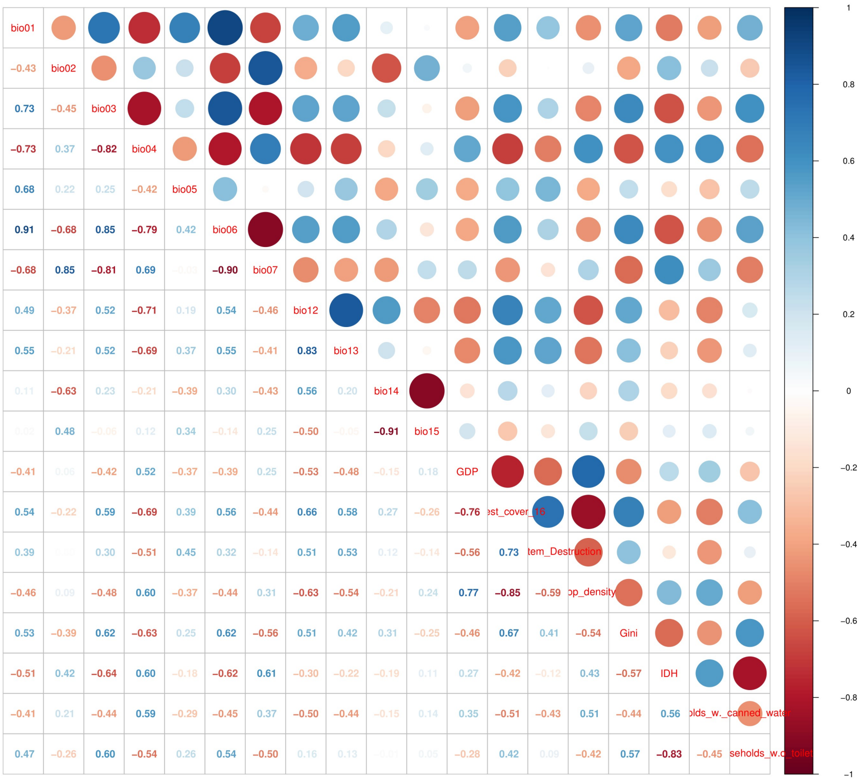

Some very interesting correlations.

Now we going choose the smallest number of variables as possible, in order to raise predictive power:

##First the environmental variables -Worldclim's variables: bio 01 (annual mean temperature) and bio 12 (annual precipitation)

-Ecosystem destruction between 2009 and 2018 (Mapbiomas- https://mapbiomas.org/) #Ecosystem destruction, or Forest and savannah loss, was highly correlated with forest cover so we decided to keep only Ecosystem destruction.

##Second socioeconomic variables:

- Mean Gross domestic product per capta (Kummu et al 2018; https://doi.org/10.1038/sdata.2018.4) highly correlated with population density,
- Gini index for inequality (IBGE 2010) and
- Proportion of households without toilet by municipality (IBGE 2010) highly correlated to IDH and as it captures a phenomenon very similar to proportion of house holds with canned water we decided to keep proportion of households without toilet.
- Socioecomic variables that were originally addressed to municipality level we-re resampled or reprojected to ~18 km cell or 10' resolution to match the others variables resolution. This process was made using 'projectRaster' function from raster package (https://cran.r-project.org/web/packages/raster/index.html) using the 'ngb'or nearest neighbor method.

```
names(var)
```

```
##  [1] "bio01"                          "bio02"
##  [3] "bio03"                          "bio04"
##  [5] "bio05"                          "bio06"
##  [7] "bio07"                          "bio12"
##  [9] "bio13"                          "bio14"
## [11] "bio15"                          "GDP"
## [13] "Forest_cover_16"                "Ecosystem_Destruction"
## [15] "Pop_density"                    "Gini"
## [17] "IDH"                            "prop_households_w._canned_water"
## [19] "prop_households_w.o_toilet"
```

```r
var_dng3<-var[[c(1,8,12,14,16, 19)]]
names(var_dng3)
```

```
## [1] "bio01"                        "bio12"
## [3] "GDP"                          "Ecosystem_Destruction"
## [5] "Gini"                         "prop_households_w.o_toilet"
```

```r
plot(var_dng3)
```

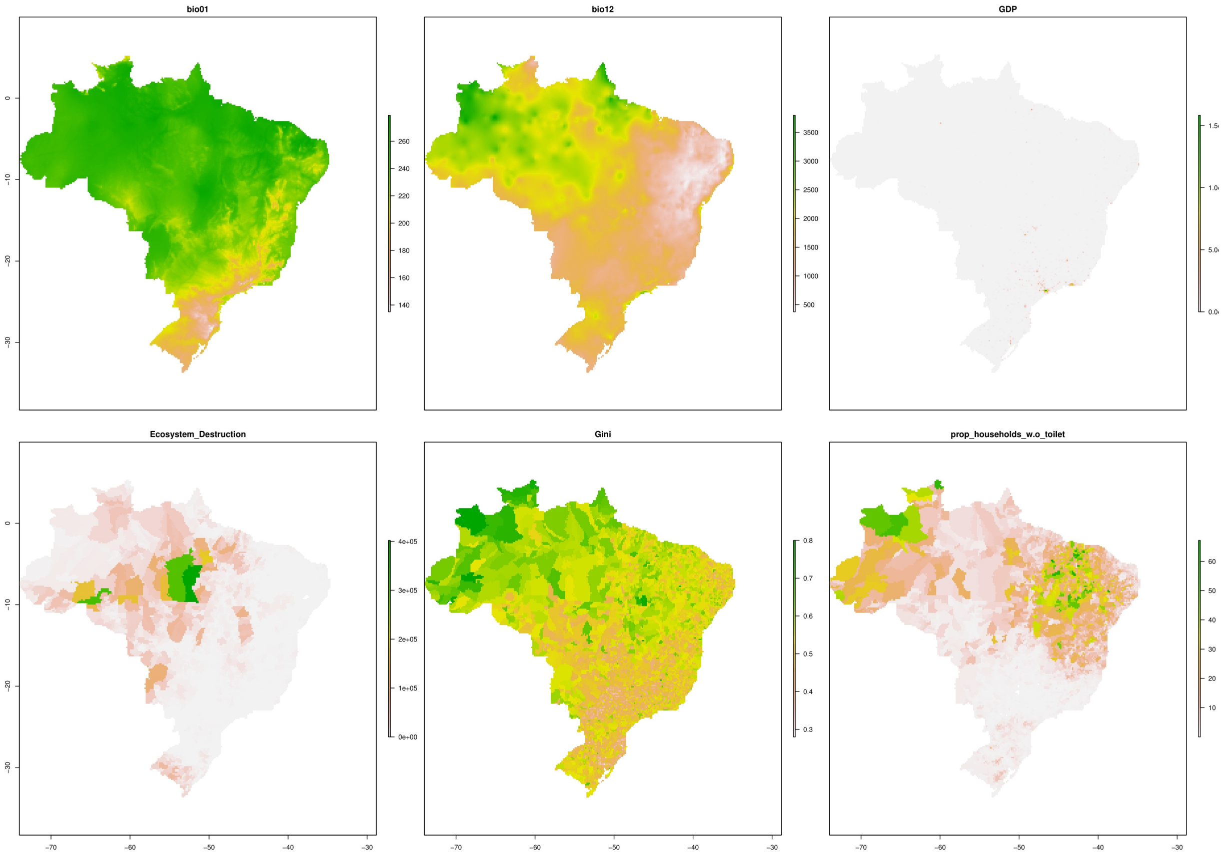


```r
var.da_dng3 <- var_dng3 %>%
  raster::values() %>%
  na.omit
corr <- cor(var.da_dng3, method = "spearman") #
corrplot::corrplot.mixed(corr, tl.srt = 45, mar = c(3, 0.5, 2, 1))
```

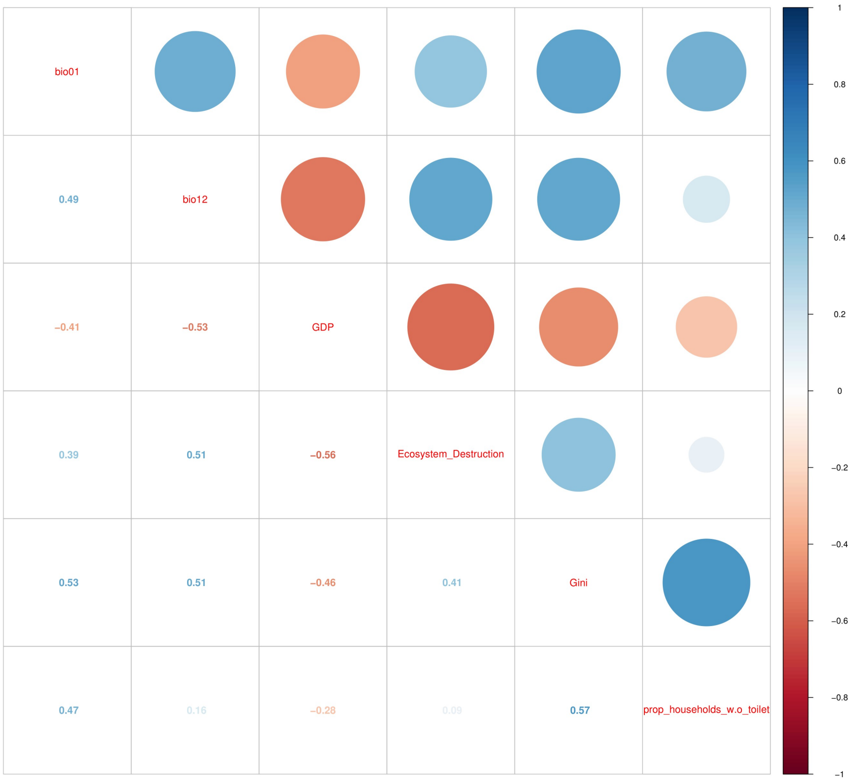

Preparing the data for a cross-validation

Sorting 500 municipalities for CV

```
mun32<- read.csv("municipios.csv")#municipalities coordinates from Brazil
mun34 <- mun32[, c(3,4)]
mun35<-cbind(mun34[,2], mun34[,1])#adjusting for ENMeval
set.seed(31)
bg_mun<-mun35[sample(nrow(mun35), 500), ]#sorting 500 muncipalities randomly
colnames(bg_mun)<-c("lon", "lat")
plot(var_dng3[[1]])+points(bg_mun)
```

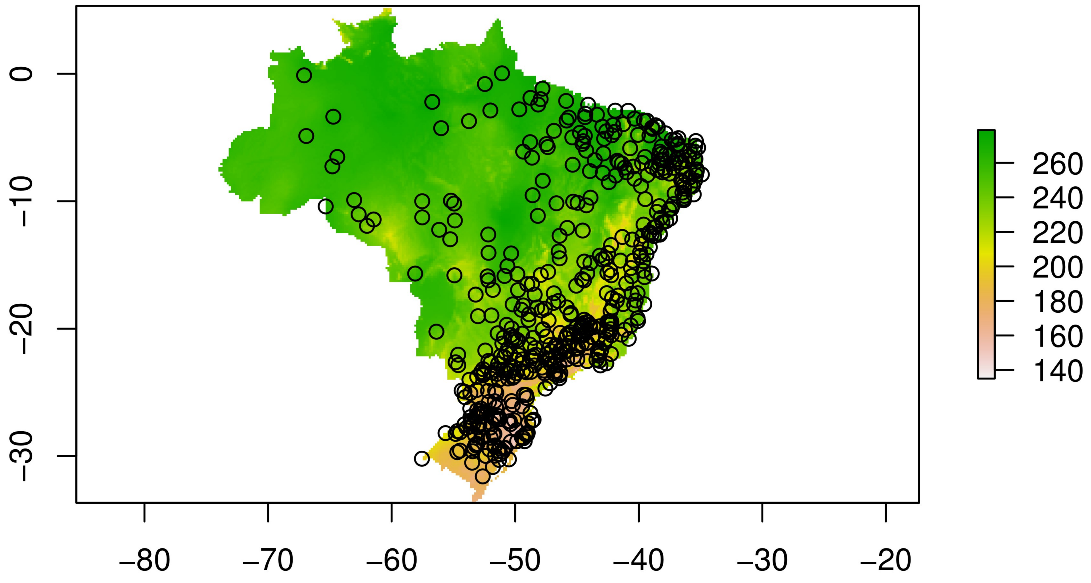


```
## integer(0)
```

```
#deviding the occurrences in 4 blocks using ENMeval
block_dng2<-get.block(dng2, bg_mun)
str(block_dng2)
```

```
## List of 2
##  $ occs.grp: num [1:3098] 1 1 1 1 1 1 1 1 1 1 ...
##  $ bg.grp  : num [1:500] 3 3 3 3 3 3 3 3 3 3 ...
```

```
plot(var_dng3[[1]],col='gray')+ points(dng2, pch=21, bg=block_dng2$occ.grp)
```

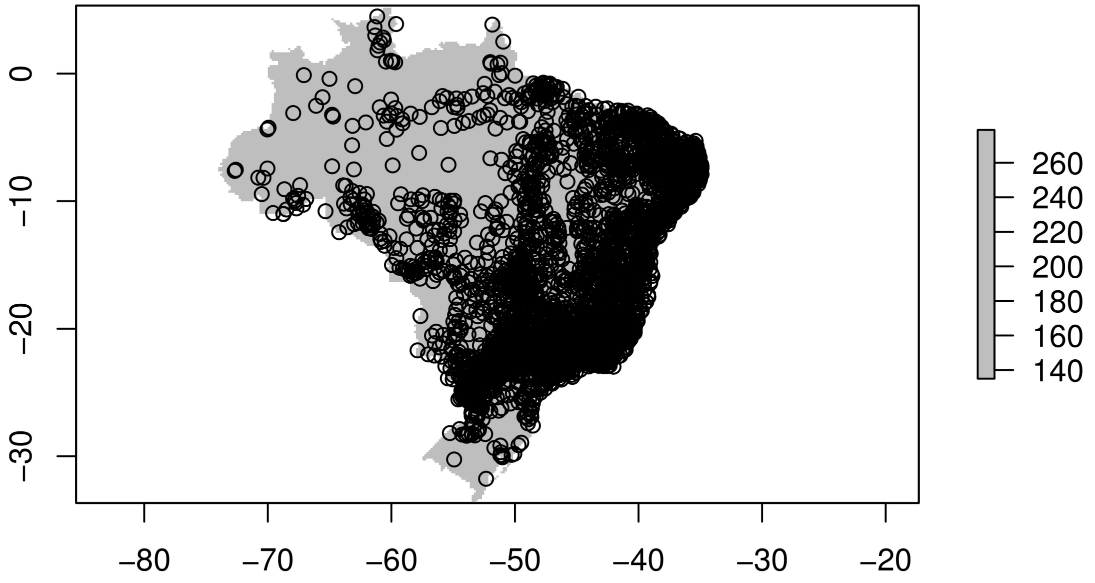


```
## integer(0)
```

Dividing the occurrences in four different blocks so we can run a cross validation H-block.

Running the models

First preparing the data for model run

```
dng3<-as.data.frame(cbind(dng2$species, dng2$lon, dng2$lat))
head(dng3)
```

```
##   V1       V2        V3
## 1  1 -61.9953 -11.92830
## 2  1 -63.0325  -9.90571
## 3  1 -60.5520 -13.49450
## 4  1 -61.4562 -11.43430
## 5  1 -60.8168 -13.18700
## 6  1 -60.5454 -13.11740
```

```
colnames(dng3)<-c("sp_name", "longitude", "latitude")
dng3$sp_name<-"dengue_fever"
dng4<-dng3[,-1]
```

```
head(dng2)
```

```
##   species
## 1       1
## 2       1
## 3       1
## 4       1
## 5       1
## 6       1
```

```
set.seed(31)#seting aleatory number for reproducibility.
d_dng<-sdmData(species~., dng2, predictors=var_dng3, bg=block_dng2$bg.grp)
```

#building the model In order to build the Ecological niche models we used the sdm package from Naimi et al. (2016; doi: 10.1111/ecog.01881) We used different algorithms like glm, maxent, bioclim, random forest and svm to build the models.

```
set.seed(31)#we set a seed to certify that random number generations will be the same thus, we can repr
m_dng<-sdm(species~.,  d_dng,methods=c('glm','maxent','bioclim', 'rf', 'svm'),#algorithms used in the m
            replication='cv', #setting method of replication for cross validation
            test.p=30, #set 30% of records for test
            cv.folds=11, #number of folds in cross validation
            n=5,#number of recplicates per algorithm
            parallelSettings=list(ncore=4, method='parallel')) #replication - specify which replication
```

```
## Carregando pacotes exigidos: parallel
```

```
m_dng
```

```
## class                                 : sdmModels
## ========================================================
## number of species                     :  1
## number of modelling methods           :  5
## names of modelling methods            :  glm, maxent, bioclim, rf, svm
## replicate.methods (data partitioning) :  cross_validation
## number of replicates (each method)    :  5
## toral number of replicates per model  :  15 (per species)
## ------------------------------------------
## model run success percentage (per species)  :
## ------------------------------------------
## method          species
## ----------------------
## glm        :        100   %
## maxent     :        100   %
## bioclim    :        100   %
## rf         :        100   %
## svm        :        100   %
##
## ###################################################################
## model Mean performance (per species), using test dataset (generated using partitioning):
## -------------------------------------------------------------------------------
```

```
##
##  ## species   :  species
## =========================
##
## methods    :     AUC     |     COR     |     TSS     |     Deviance
## -----------------------------------------------------------------------------
## glm        :     0.79    |     0       |     0.62    |     0.09
## maxent     :     NaN     |     NaN     |     NaN     |     NaN
## bioclim    :     0.61    |     0.01    |     0.44    |     7.41
## rf         :     0.82    |     0.21    |     0.66    |     0.01
## svm        :     0.58    |     0.08    |     0.58    |     0.02
```

Individual predictions from algorithms

Since the model is done let's have a look in the individual predictions.

```
dng_p1<-predict(m_dng, var_dng3, filename='dng_sdm5.img', overwrite=TRUE)
```

```
plot(dng_p1)
```

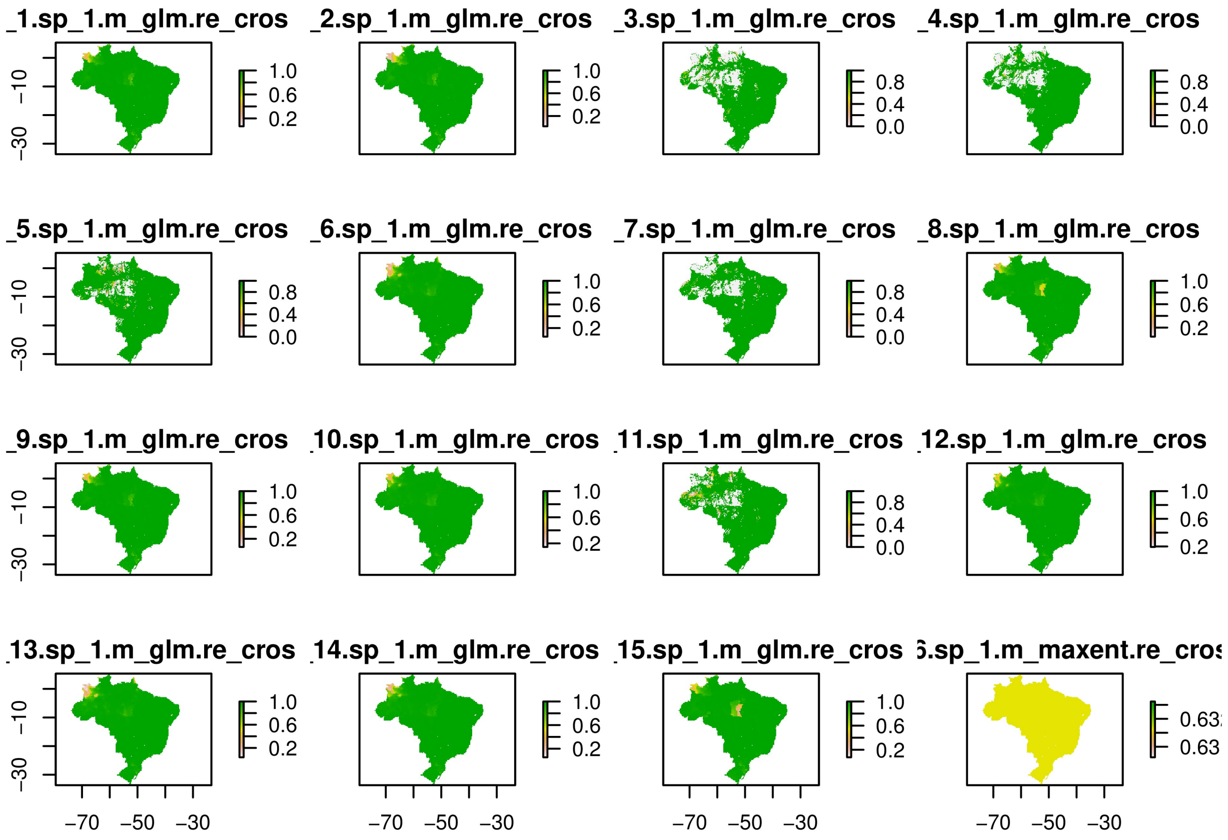

Plotting at least one prediction of each algorithm to have a closer look…

```r
plot(dng_p1[[c(1,7,15,16,21,30,31,37,45,46,53,60,61,68,75)]])
```

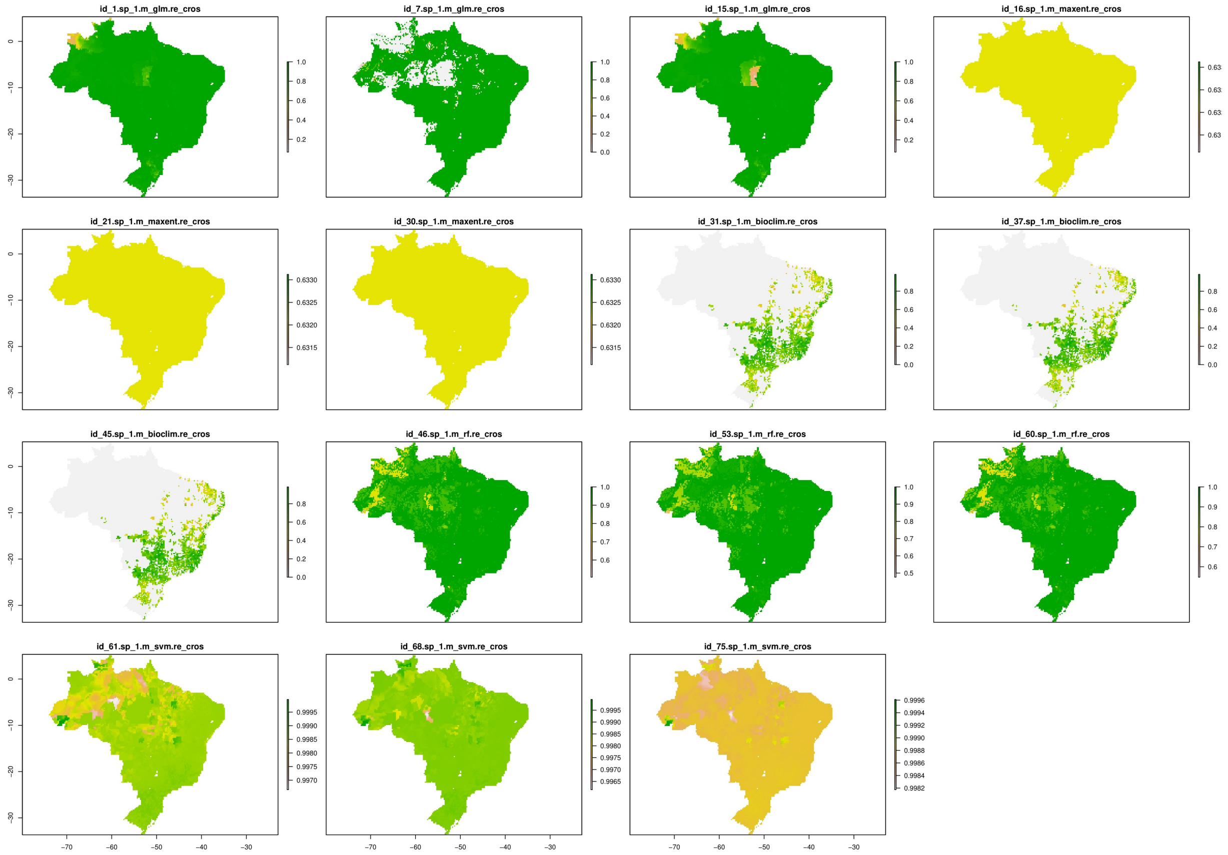


```r
#have a look in all algorithms
```

Now binarizing these continuous models using niche tool box.

We can check if the prediction is informative

```r
dng_bin1 <- bin_model(dng_p1[[1:15]], dng2,percent = 10)#percent is the threshold
dng_bin2 <- bin_model(dng_p1[[16:30]],dng2,percent = 10)
dng_bin3 <- bin_model(dng_p1[[31:45]],dng2,percent = 10)
dng_bin4 <- bin_model(dng_p1[[46:60]],dng2,percent = 10)
dng_bin5 <- bin_model(dng_p1[[61:75]],dng2,percent = 10)
#and now cheking each prediction
```

```r
plot(dng_bin1)#glm
```

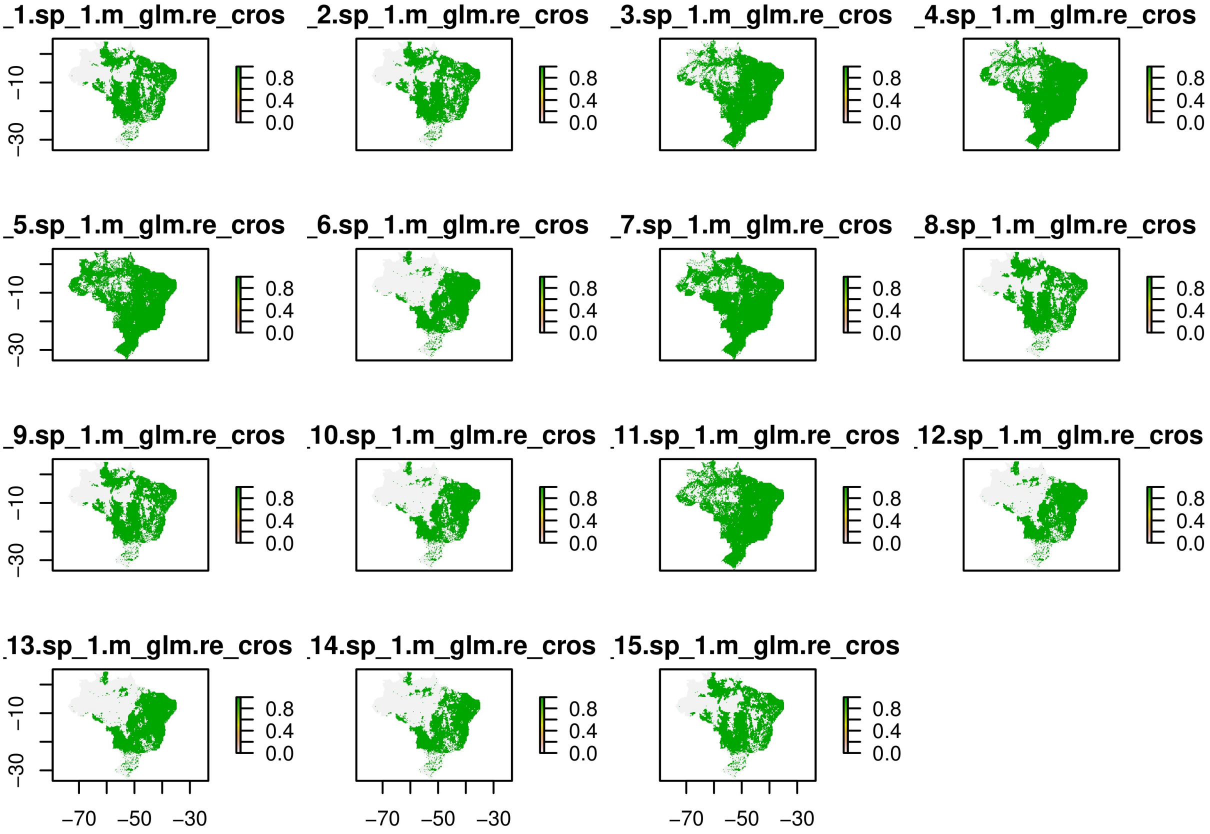


```
plot(dng_bin2)#maxent
```

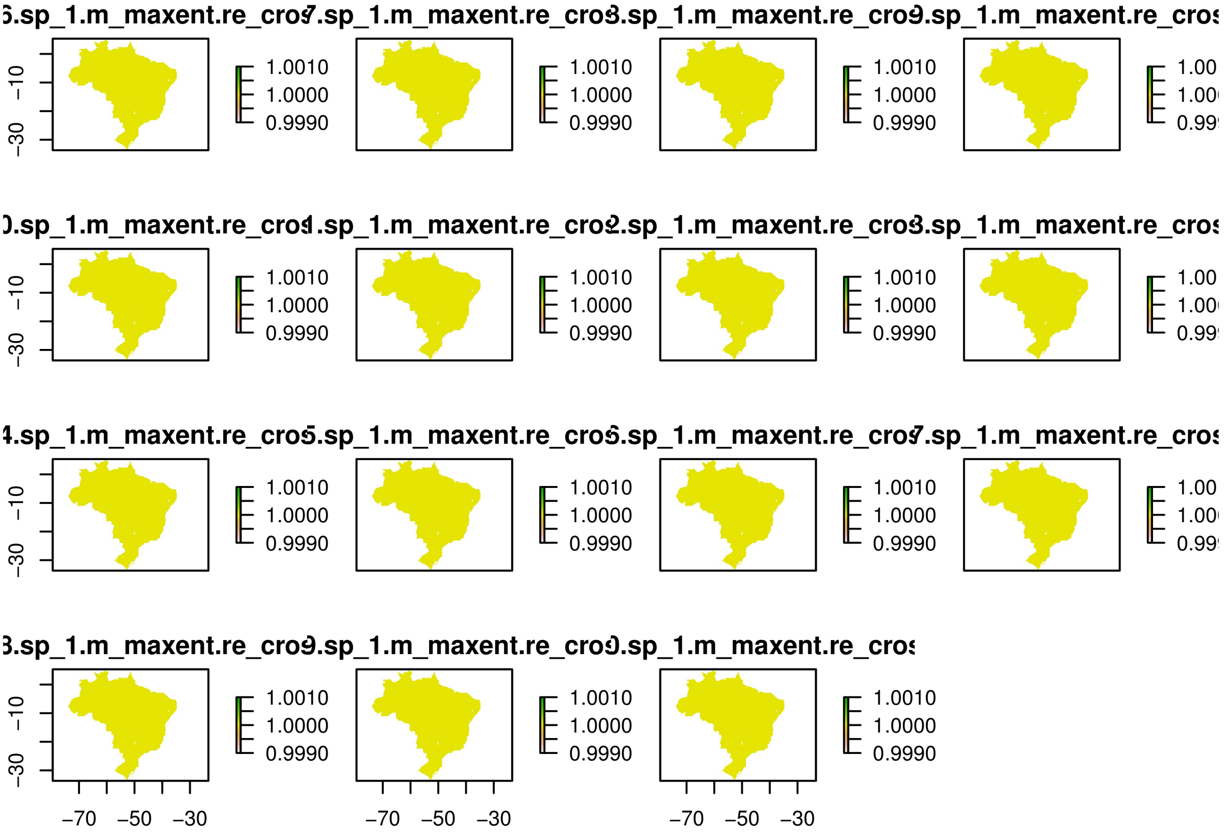


```
plot(dng_bin3)#bioclim
```

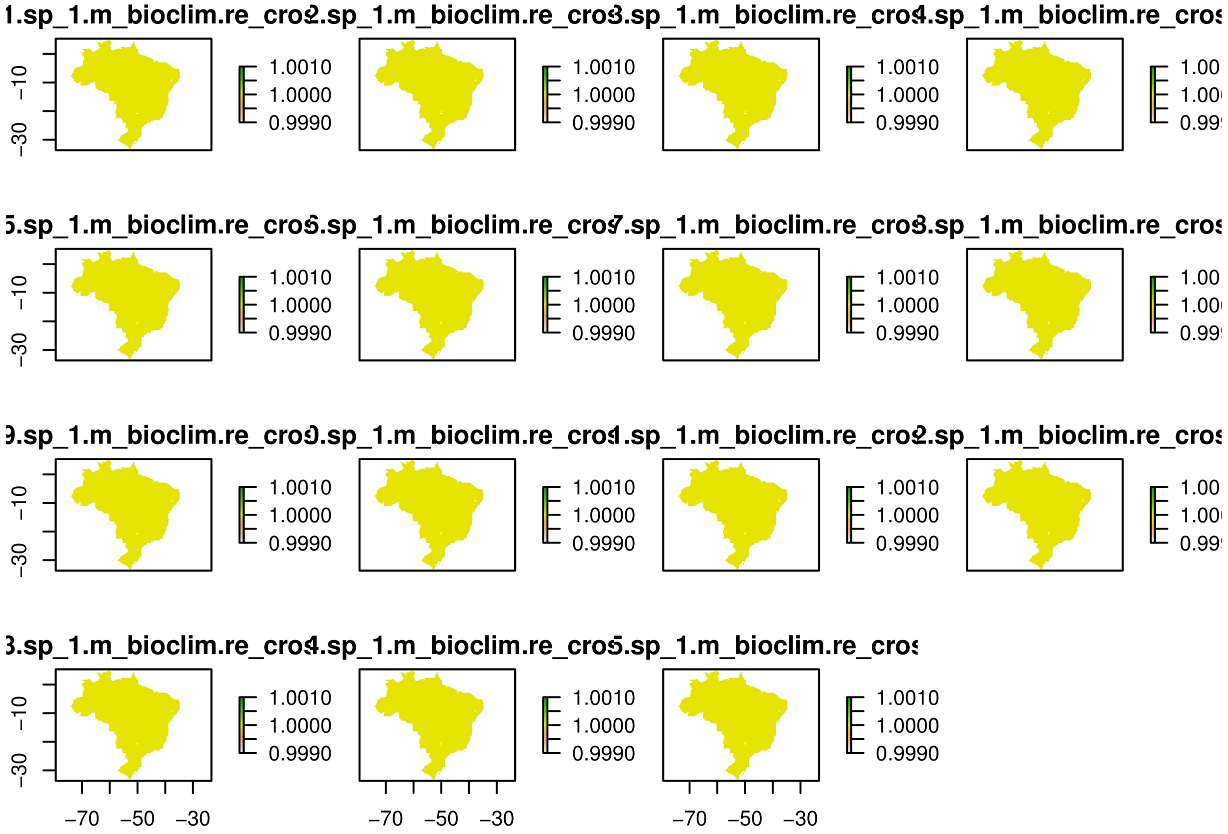


```
plot(dng_bin4)#Random Forest
```

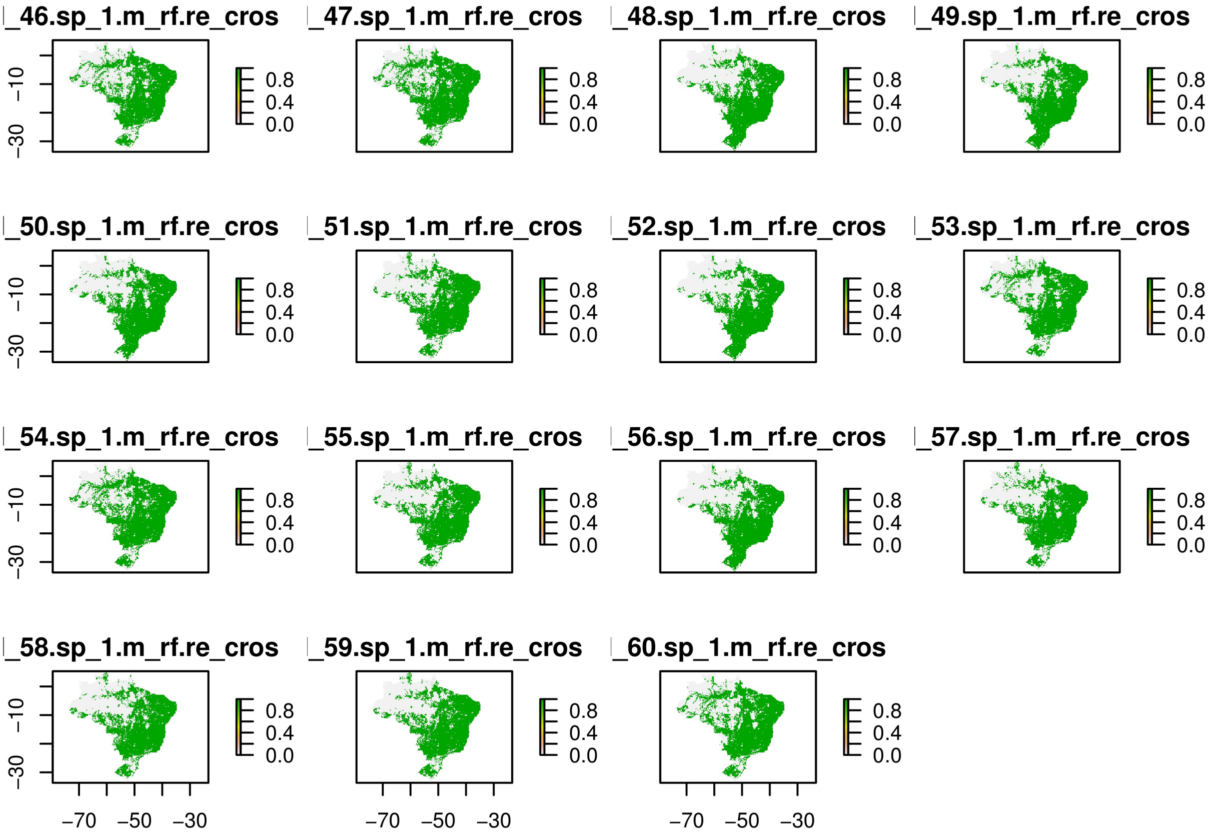


```
plot(dng_bin5)#SVM
```

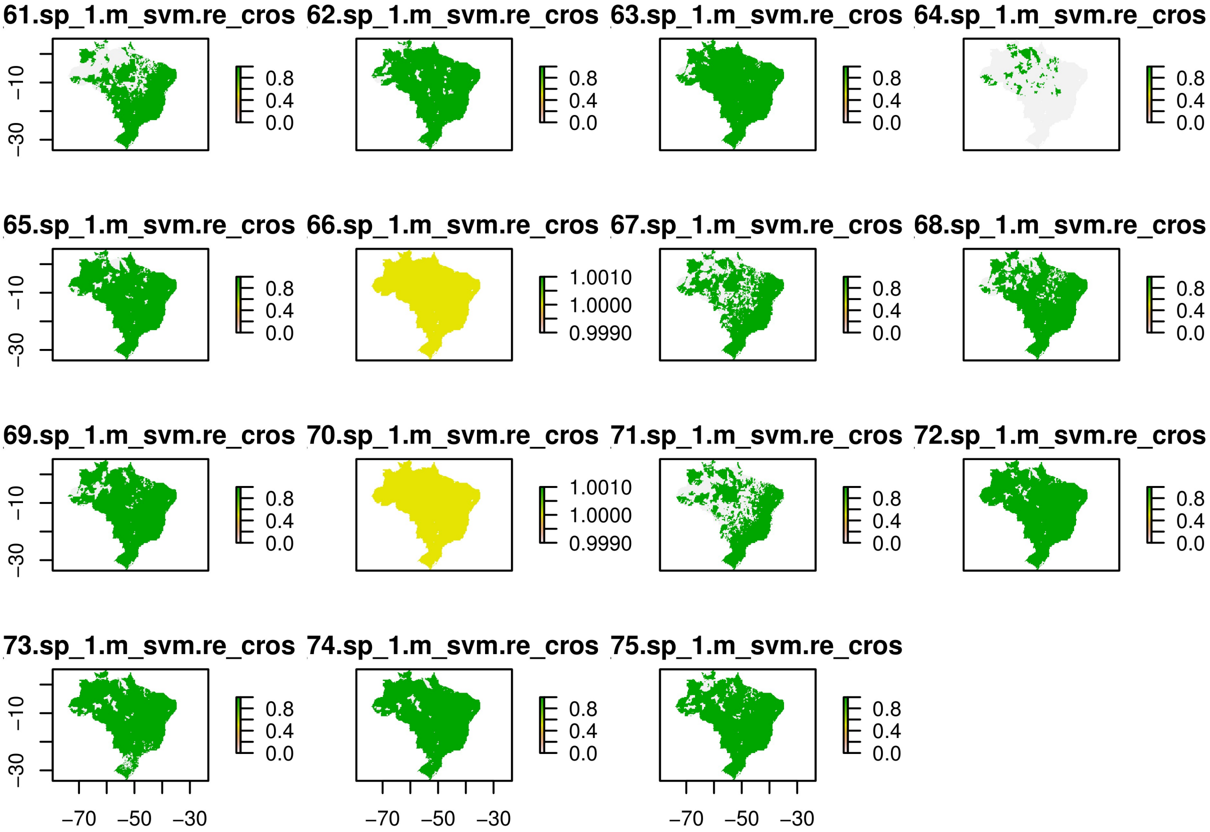

It's a bit dificult to look so let's plot 2 of each algorithm

```
dng_binary<-raster::stack(dng_bin1,
                           dng_bin2,
                           dng_bin3,
                          dng_bin4,
                          dng_bin5)
plot(dng_binary[[c(1,7,15,16,21,30,31,37,45,46,53,60,61,68,75)]])
```

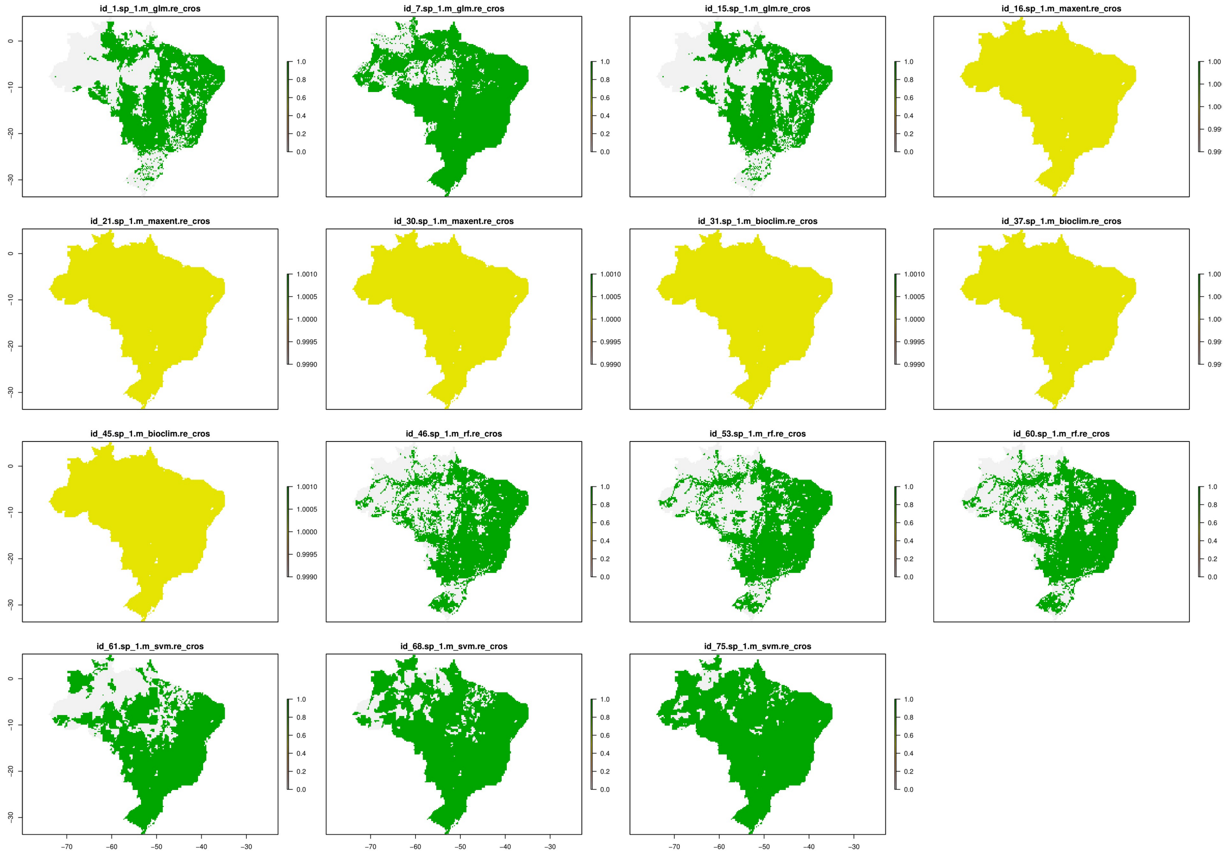


```
#have a look in all algorithms
```

Bioclim and Maxent did not bring any information after binarization (Maxent did not bring information before binarization either).

So we decided to re-run dengue models without these two algorithms. ##Model Re-run Just repeat the steps so far without Maxent and Bioclim

```
set.seed(31)
m_dng<-sdm(species~.,  d_dng,methods=c('glm', 'rf', 'svm', 'gam'),
           replication='cv',
           test.p=30,
           cv.folds=11,
           n=5,
           parallelSettings=list(ncore=4, method='parallel'))
dng_p1<-predict(m_dng, var_dng3, filename='dng_sdm4.img', overwrite=TRUE)
dng_bin1 <- bin_model(dng_p1[[1:15]], dng2,percent = 10)#glm
dng_bin2 <- bin_model(dng_p1[[16:30]],dng2,percent = 10)#random forest
dng_bin3 <- bin_model(dng_p1[[31:45]],dng2,percent = 10)#svm
dng_bin4 <- bin_model(dng_p1[[46:60]],dng2,percent = 10)#gam
plot(dng_bin4)
```

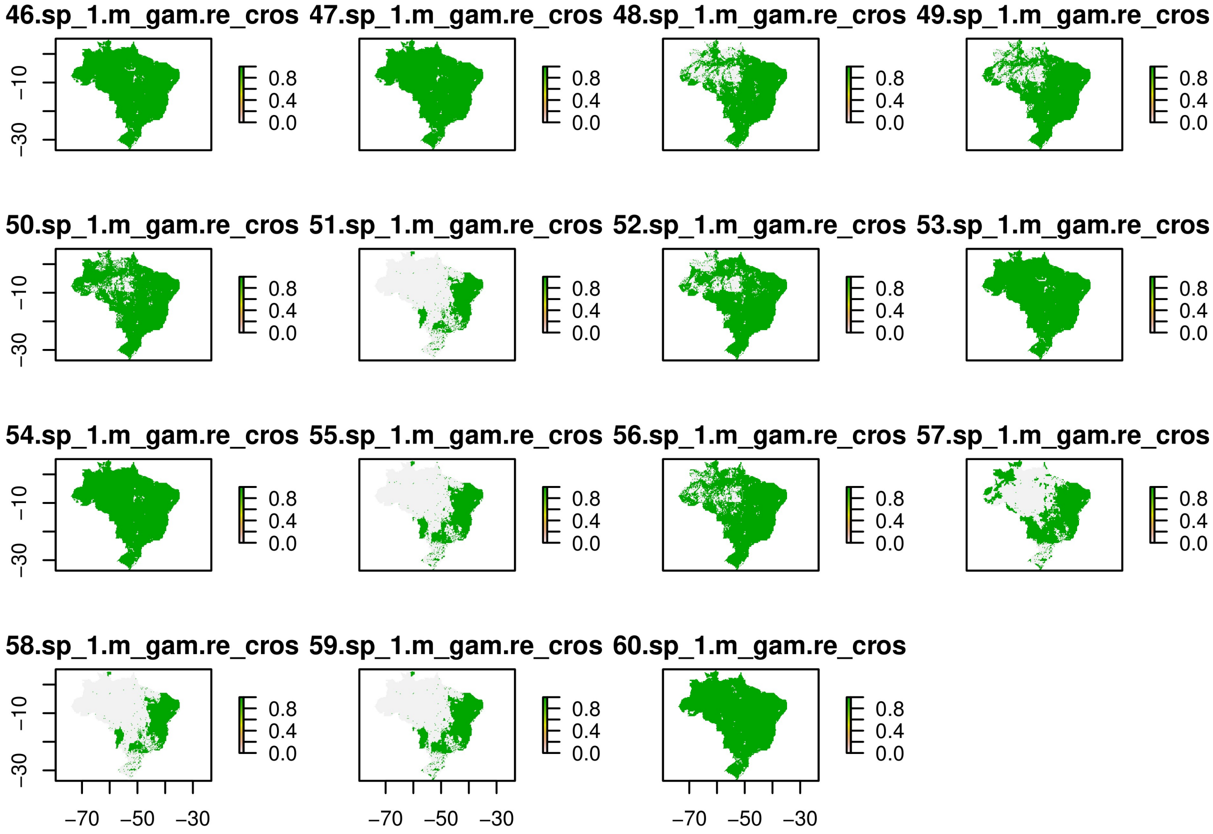


```
dng_binary<-raster::stack(dng_bin1,
                           dng_bin2,
                           dng_bin3, dng_bin4)
```

#Now the individual models are ready for ensemble ##Building the socioeconomic + environemnt ensemble model

```
dng_p2_en2 <- ensemble(m_dng, dng_binary, filename='en_dng_sdm_binary35.tif', setting=list(method='unwe
```

```
##
##  ......... the Raster object is used as the predicted probabilities...
```

```
#'method='unweighted' - uses a ensemble strategies withouth weights. In this way, the suitability value
plot(dng_p2_en2, col=matlab.like2(100))
```

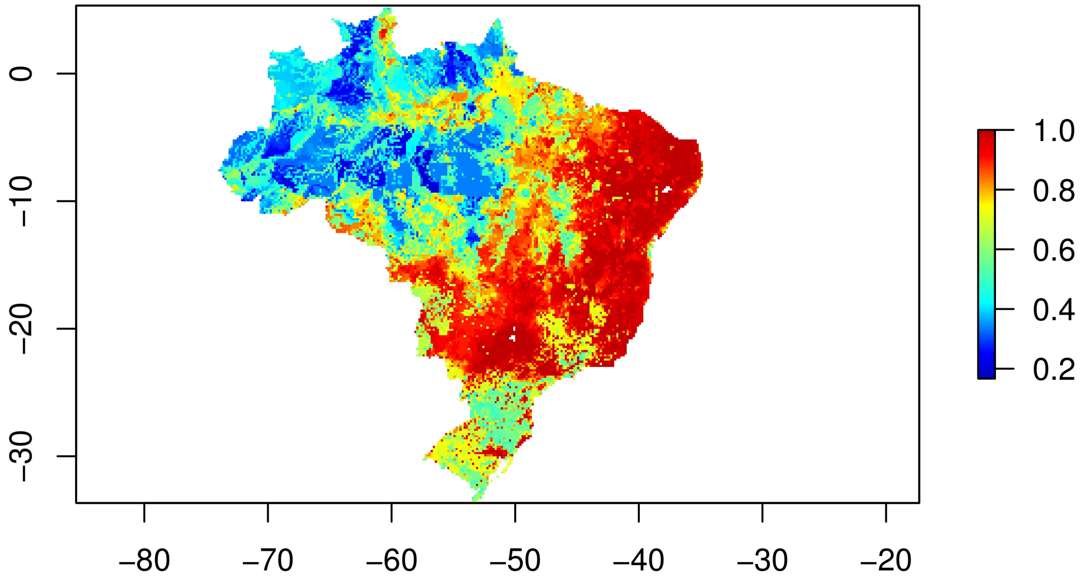

Plotting it with occurrences

```
plot(dng_p2_en2, col=matlab.like2(100))+points(dng2)
```

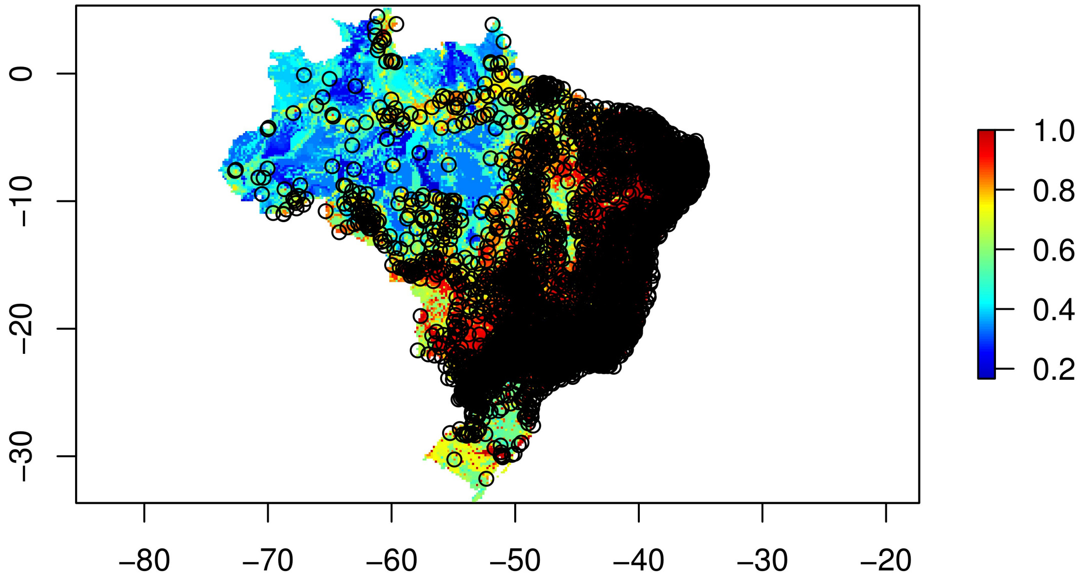


```
## integer(0)
```

Plotting in a good looking pallet of colors

```
dng_viridis<-as.data.frame(dng_p2_en2, xy=T)
head(dng_viridis)
```

```
##          x    y en_dng_sdm_binary35
## 1 -73.91667 5.25                  NA
## 2 -73.75000 5.25                  NA
## 3 -73.58333 5.25                  NA
## 4 -73.41667 5.25                  NA
## 5 -73.25000 5.25                  NA
## 6 -73.08333 5.25                  NA
```

```
dng_test1<-ggplot() +
  geom_raster(data = subset(dng_viridis, !is.na(en_dng_sdm_binary35)), aes(x=x, y=y, fill=en_dng_sdm_bi
              alpha=0.8)+
  scale_alpha(range =  c(0.15, 0.65), guide = "none") +
  scale_fill_viridis_c()+
  theme_bw()+
  labs(x=NULL, y=NULL, fill="Disease risk (suitability)")+
  coord_quickmap()
dng_test1
```

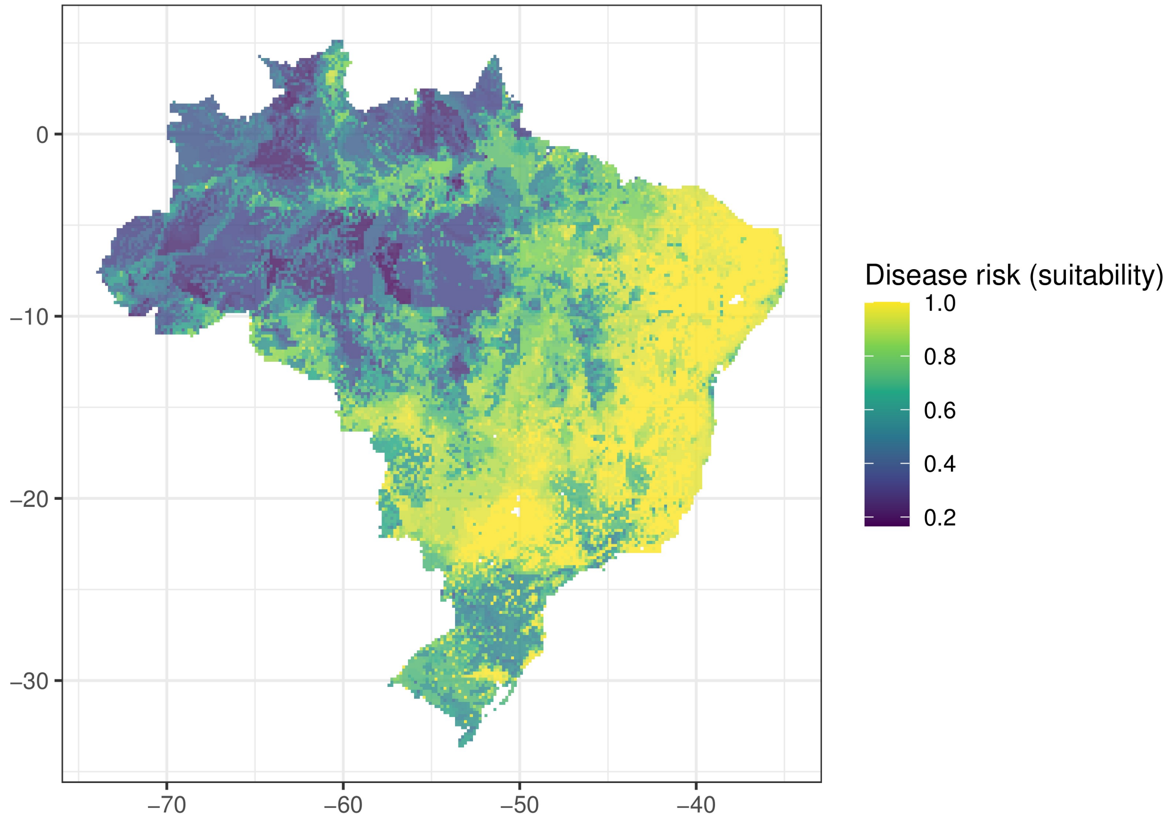


```
dng_test11<-dng_test1+theme(panel.grid.major = element_blank(), panel.grid.minor = element_blank())
dng_test11+geom_point(data = dng3,
           aes(x = longitude,
               y = latitude,
               color=sp_name),
           shape=1,
           size = 2)+
  scale_shape_manual(values = 1) +
  scale_color_manual(values = "#FC4E07")+
  labs(color="Disease Occurrences", shape=NULL)
```

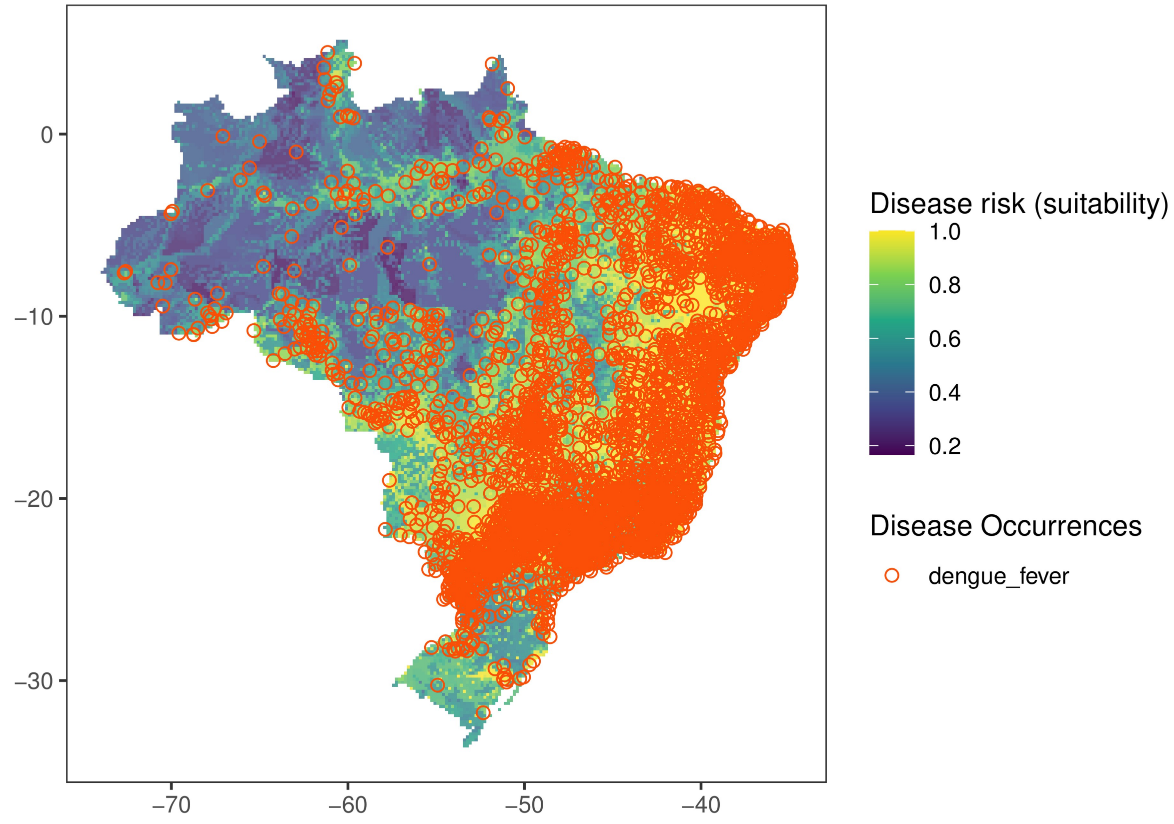

#Partial Roc evaluation Now performing the partial Roc, with pROC function from 'ntbox' package (Osorio-Olvera et al. 2020; https://doi.org/10.1111/2041-210X.13452) The partial ROC metrics were applied using ntbox R package to 50% subset of presence data, based on 1000 iterations and using a 5% error threshold (all default arguments).

```
pROC_dng <- pROC(continuous_mod=dng_p2_en2,
                 test_data=dng4,
                 n_iter=1000,
                 E_percent=5,
                 boost_percent=50,
                 parallel=FALSE)

pROC_dng$pROC_summary
```

```
##              Mean_AUC Mean_pAUC_ratio_at_5%               P_value
##             0.7910301             1.2763538             0.0000000
```

Now ploting the relative variable importance using the function 'getVarImp' from sdm package (Naimi et al. 2016) from the model. This informs the variables that are most important for defining the model.

```
plot(getVarImp(m_dng))
```

```
##
## The variable importance for all the models are combined (averaged)...
```

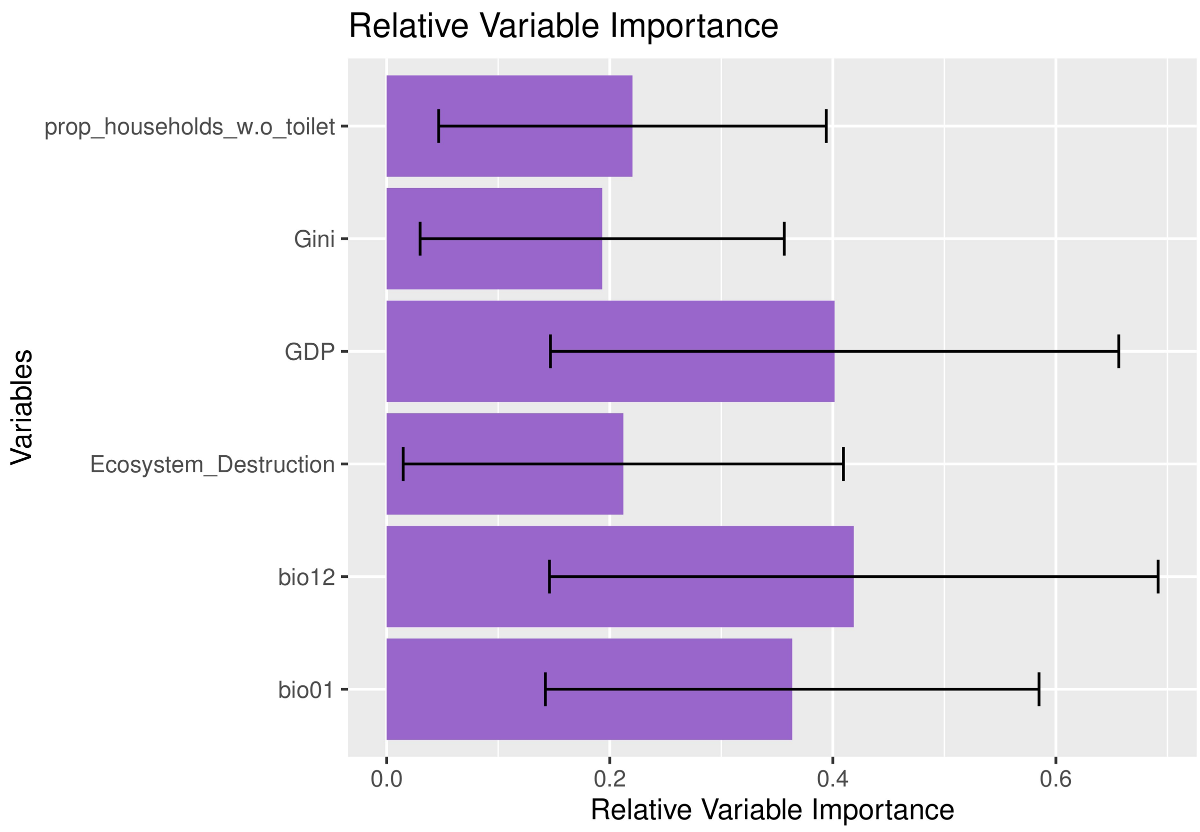

Response curves area other way to explore variable importance, in order to visualize how each variable responds to the response variable, in this case disease suitability. (this procedure is implemented in sdm package, following Elith et al. 2005; https://doi.org/10.1016/j.ecolmodel.2004.12.007)

```
rcurve(m_dng)
```

```
## The id argument is not specified; The modelIDs of 60 successfully fitted models are assigned to id..
```

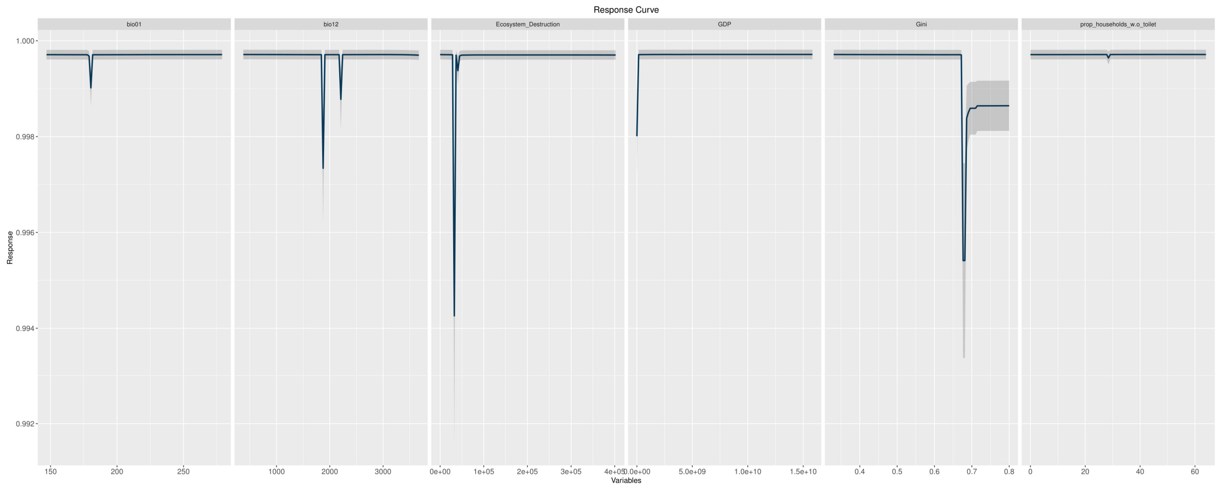

In this method response curves are strange since the models were binarized in order to promote interpretation. we can have a look each algorithm by time

```r
rcurve(m_dng, id=1:15)#glm
```

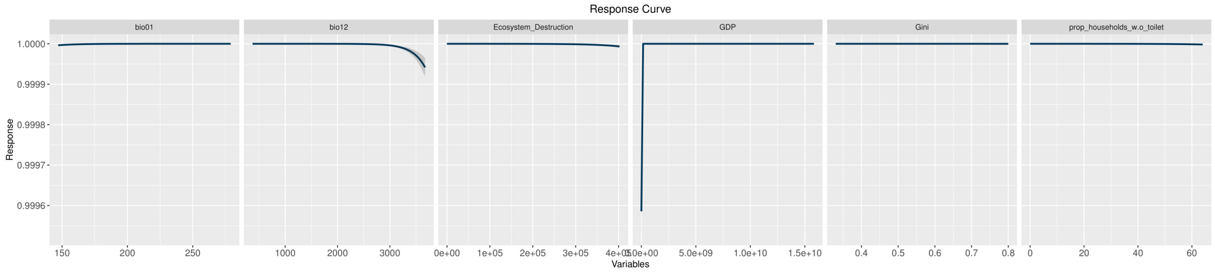


```r
rcurve(m_dng, id=16:30)#random forest
```

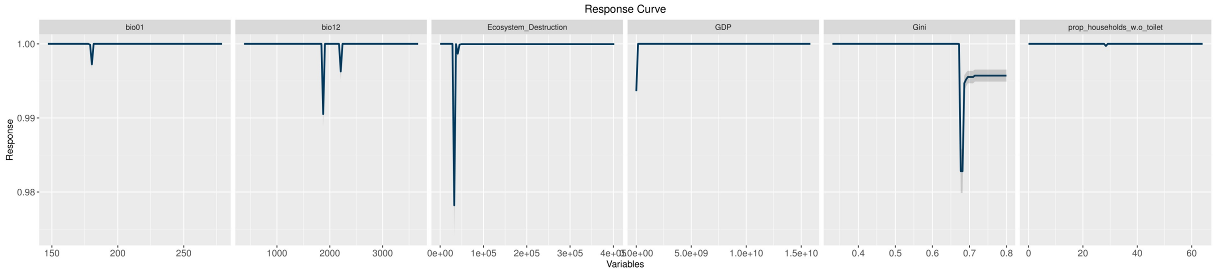


```r
rcurve(m_dng, id=31:45)#SVM
```

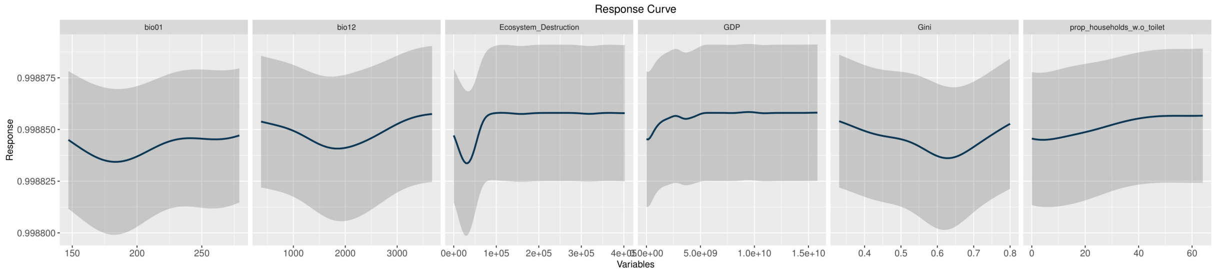

Building Environment only models

Selecting only environment relevant variables

```r
names(var)
```

```
##  [1] "bio01"                           "bio02"
##  [3] "bio03"                           "bio04"
##  [5] "bio05"                           "bio06"
##  [7] "bio07"                           "bio12"
##  [9] "bio13"                           "bio14"
## [11] "bio15"                           "GDP"
## [13] "Forest_cover_16"                 "Ecosystem_Destruction"
## [15] "Pop_density"                     "Gini"
## [17] "IDH"                             "prop_households_w._canned_water"
## [19] "prop_households_w.o_toilet"
```

```r
var_clim<-var[[c(1:11,14)]]
plot(var_clim)
```

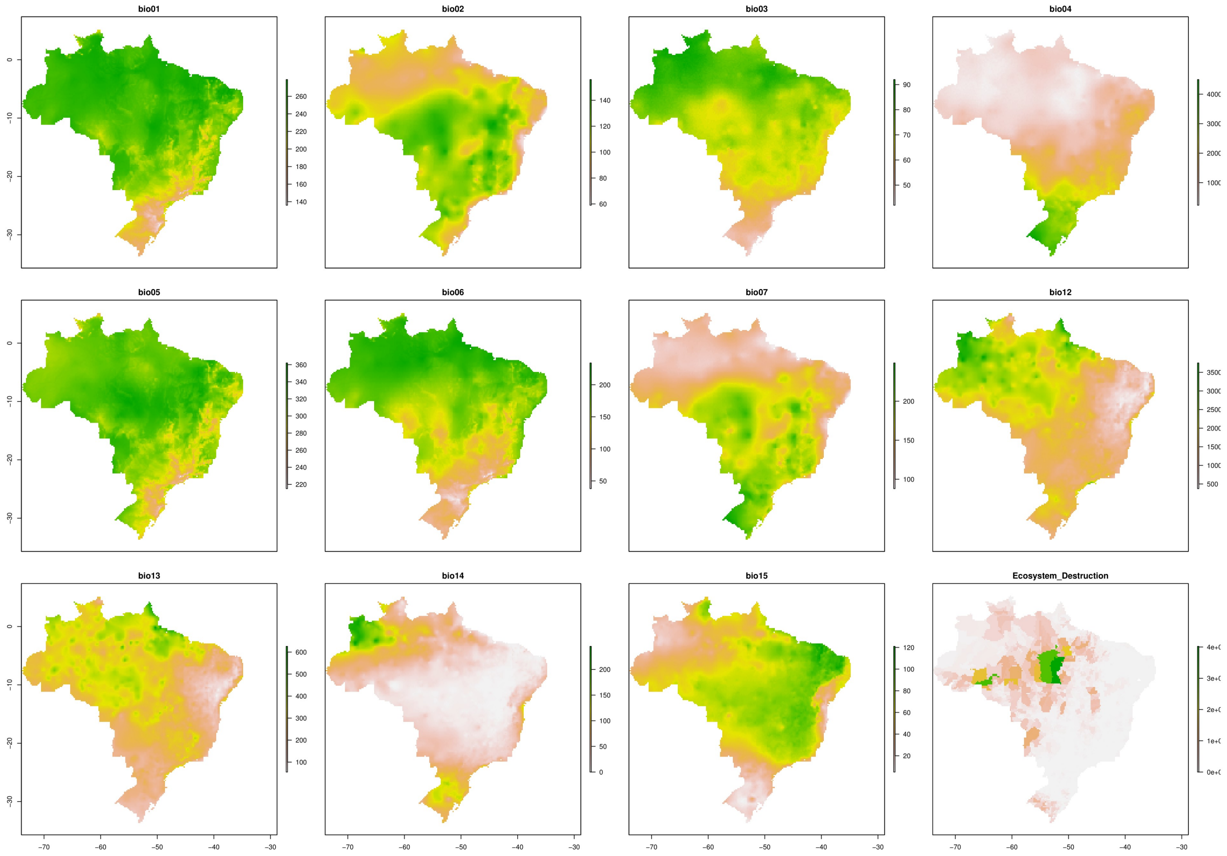


```r
names(var_clim)
```

```
##  [1] "bio01"                  "bio02"                  "bio03"
##  [4] "bio04"                  "bio05"                  "bio06"
##  [7] "bio07"                  "bio12"                  "bio13"
## [10] "bio14"                  "bio15"                  "Ecosystem_Destruction"
```

```r
var.da_clim <- var_clim %>%
  raster::values() %>%
  na.omit
corr <- cor(var.da_clim, method = "spearman")
corrplot:: corrplot.mixed(corr, tl.srt = 45, mar = c(3, 0.5, 2, 1))
```

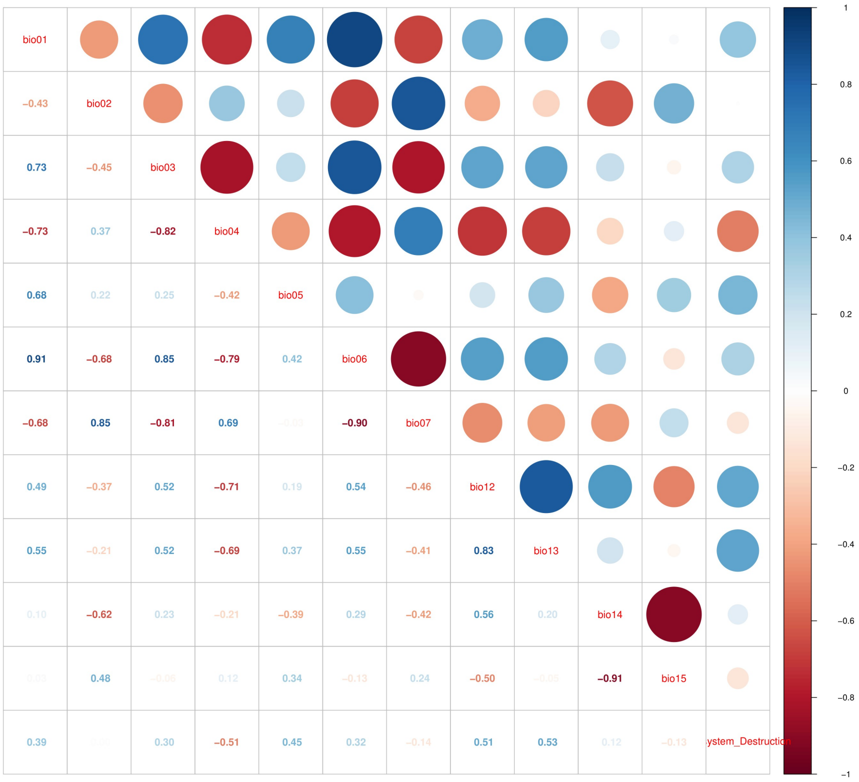

Now we will choose the variables to make a collection of just environmental variables. We sought to recreate a variable selection process that was as close to the traditional ENM approach as possible. To identify less correlated climatic variables, we utilized the 'vifstep' function from the sdm R package (61). We utilized vifstep to select them, removing variables with an overall correlation score of more than '2', which is a conservative number for colinearity. As a result, there is no colinearity among the variables, and the environmental models continue to use a set of variables that are similar to socioeconomic models (which is important since we want to compare the performance between them).

```
ex_dng_clim<-raster::extract(var_clim, dng2) #extracting records from variables among occurrences
v_dng_clim<-vifstep(ex_dng_clim, th = 2)#calculating

var_dng_clim<-exclude(var, v_dng_clim)
var.da_dng_clim <- var_dng_clim %>%
  raster::values() %>%
  na.omit
corr <- cor(var.da_dng_clim, method = "spearman")
corrplot:: corrplot.mixed(corr, tl.srt = 45, mar = c(3, 0.5, 2, 1))
```

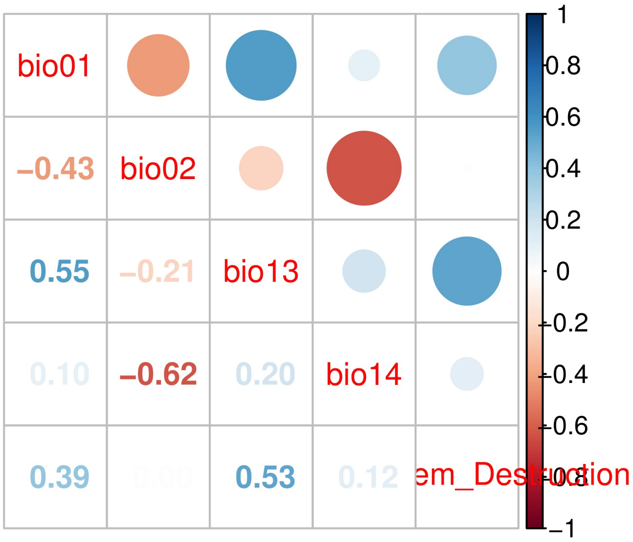

#generating the environment only models Here we will generate a new ENM using the same occurrence data and the new set of variables (var_dng_clim), performing the same cross validation as before. Furthermore, we will use the same set of algorithms:

```
set.seed(31)
d_dng_clim<-sdmData(species~.,dng2, predictors = var_dng_clim, bg=block_dng2$bg.grp)
```

```
## Warning in if (nrow(x) > size) {: a condição tem comprimento > 1 e somente o
## primeiro elemento será usado
```

```
m_dng_clim<-sdm(species~.,  d_dng_clim, methods=c('maxent','bioclim','glm', 'rf', 'svm'),
                 replication='cv',
                 test.p=30,
                cv.folds=11,
                n=5,
                 parallelSettings=list(ncore=4, method='parallel'))
```

Now repeating the same procedure of binarization and checking how individual algorithms performed:

```
dng_p1_clim<-predict(m_dng_clim, var_dng_clim, filename='model_dng_tegu_comcv.img', overwrite=TRUE)
plot(dng_p1_clim)
```

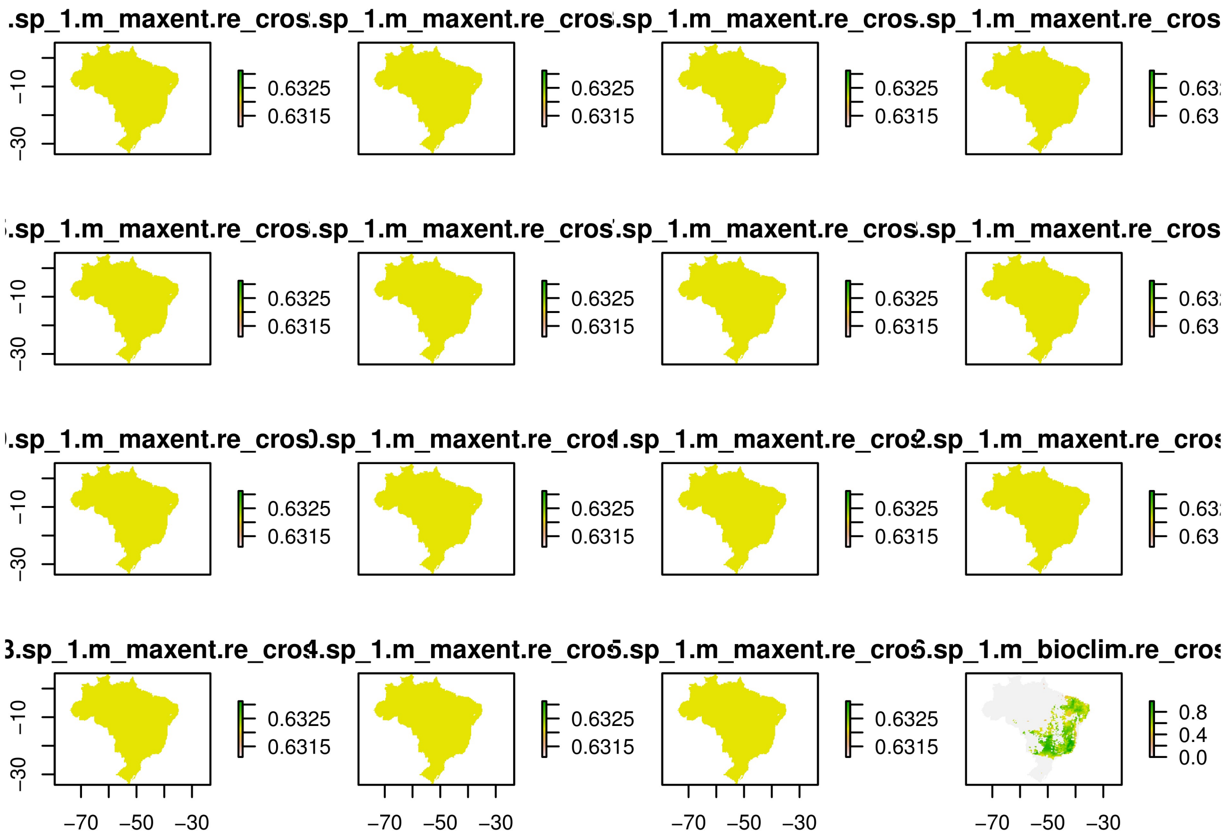


```
dng_clim_bin1 <- bin_model(dng_p1_clim[[1:15]], dng2,percent = 10)
dng_clim_bin2 <- bin_model(dng_p1_clim[[16:30]],dng2,percent = 10)
dng_clim_bin3 <- bin_model(dng_p1_clim[[31:45]],dng2,percent = 10)
dng_clim_bin4 <- bin_model(dng_p1_clim[[46:60]],dng2,percent = 10)
dng_clim_bin5 <- bin_model(dng_p1_clim[[61:75]],dng2,percent = 10)

dng_clim_binary<-raster::stack(dng_clim_bin1,
                               dng_clim_bin2,
                               dng_clim_bin3,
                               dng_clim_bin4,
                               dng_clim_bin5)
plot(dng_clim_binary[[c(1,7,15,16,21,30,31,37,45,46,53,60,61,68,75)]])
```

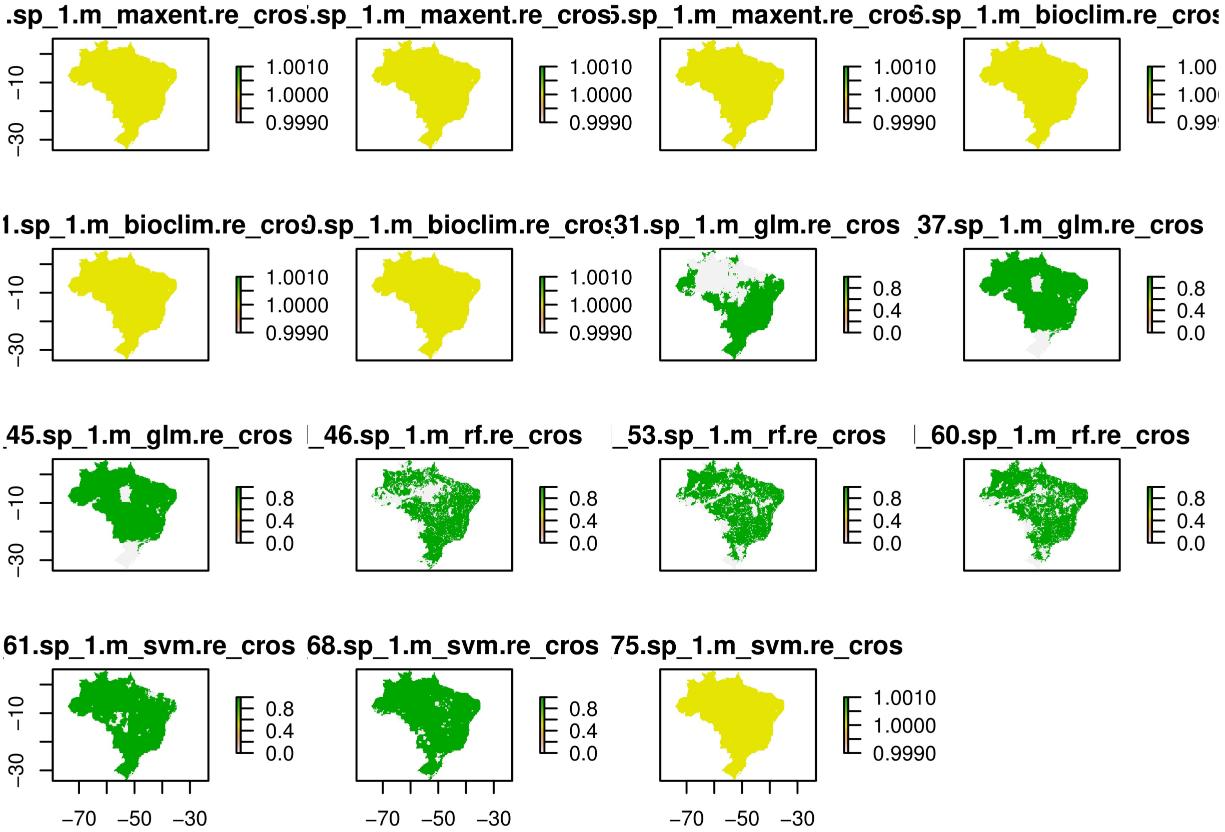

Again Bioclim and Maxent turn up to be non informative models after binarization. So we will repeat the process to generate the model without these algorithms.

```
set.seed(31)
m_dng_clim<-sdm(species~.,  d_dng_clim, methods=c('glm', 'rf', 'svm'),
                replication='cv',
                test.p=30,
                cv.folds=11,
                n=5,
                parallelSettings=list(ncore=4, method='parallel'))


dng_p1_clim<-predict(m_dng_clim, var_dng_clim, filename='model_dng_cv.img', overwrite=TRUE)

dng_clim_bin1 <- bin_model(dng_p1_clim[[1:15]], dng2,percent = 10)
dng_clim_bin2 <- bin_model(dng_p1_clim[[16:30]],dng2,percent = 10)
dng_clim_bin3 <- bin_model(dng_p1_clim[[31:45]],dng2,percent = 10)


dng_clim_binary<-raster::stack(dng_clim_bin1,
                               dng_clim_bin2,
                               dng_clim_bin3)
```

Performing again the ensemble with the mean between the remaining algorithms.

```
dng_clim_en2 <- ensemble(m_dng_clim, dng_clim_binary, filename='en_dng_clim_binary38.tif', setting=list
```

```
##
##  ......... the Raster object is used as the predicted probabilities...
```

```
plot(dng_clim_en2, col=matlab.like2(100))
```

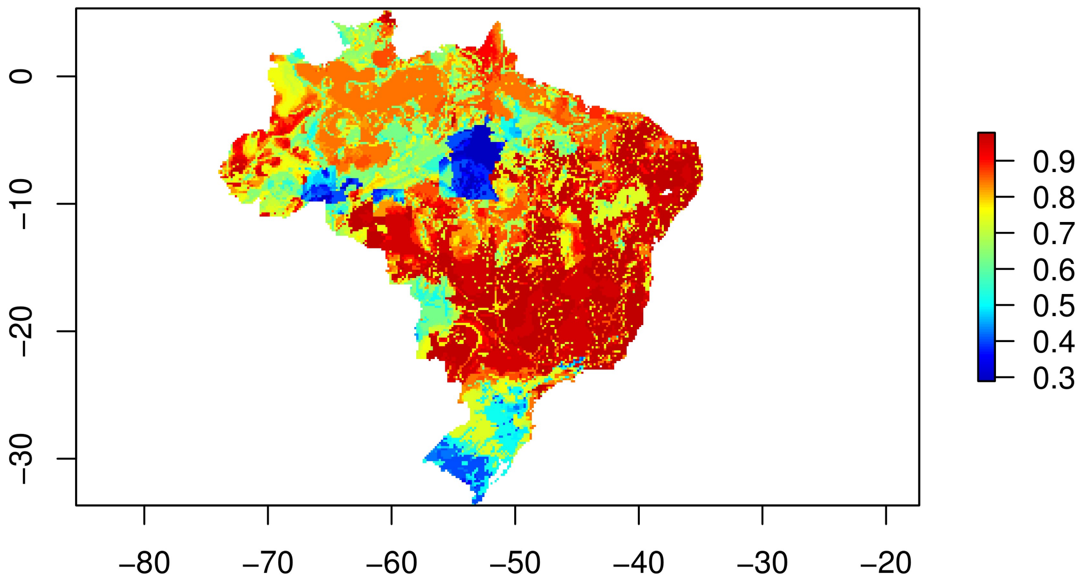


```
plot(dng_clim_en2, col=matlab.like2(100))+points(dng4)
```

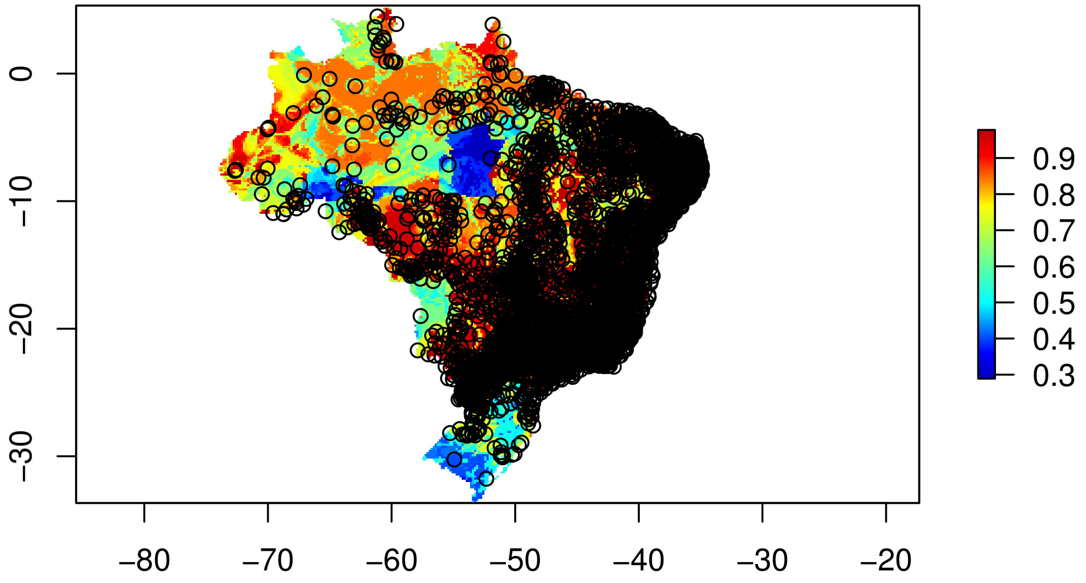


```
## integer(0)
```

Plotting the same with a different pallet of colours:

```
dng_clim_viridis<-as.data.frame(dng_clim_en2, xy=T)

dng_test2<-ggplot() +
  geom_raster(data = subset(dng_clim_viridis, !is.na(en_dng_clim_binary38)), aes(x=x, y=y, fill=en_dng_
              alpha=0.8)+

  scale_alpha(range =  c(0.15, 0.65), guide = "none") +
  scale_fill_viridis_c()+
  theme_bw()+
  labs(x=NULL, y=NULL, fill="Disease risk (suitability)")+
  coord_quickmap()

plot(dng_test2)
```

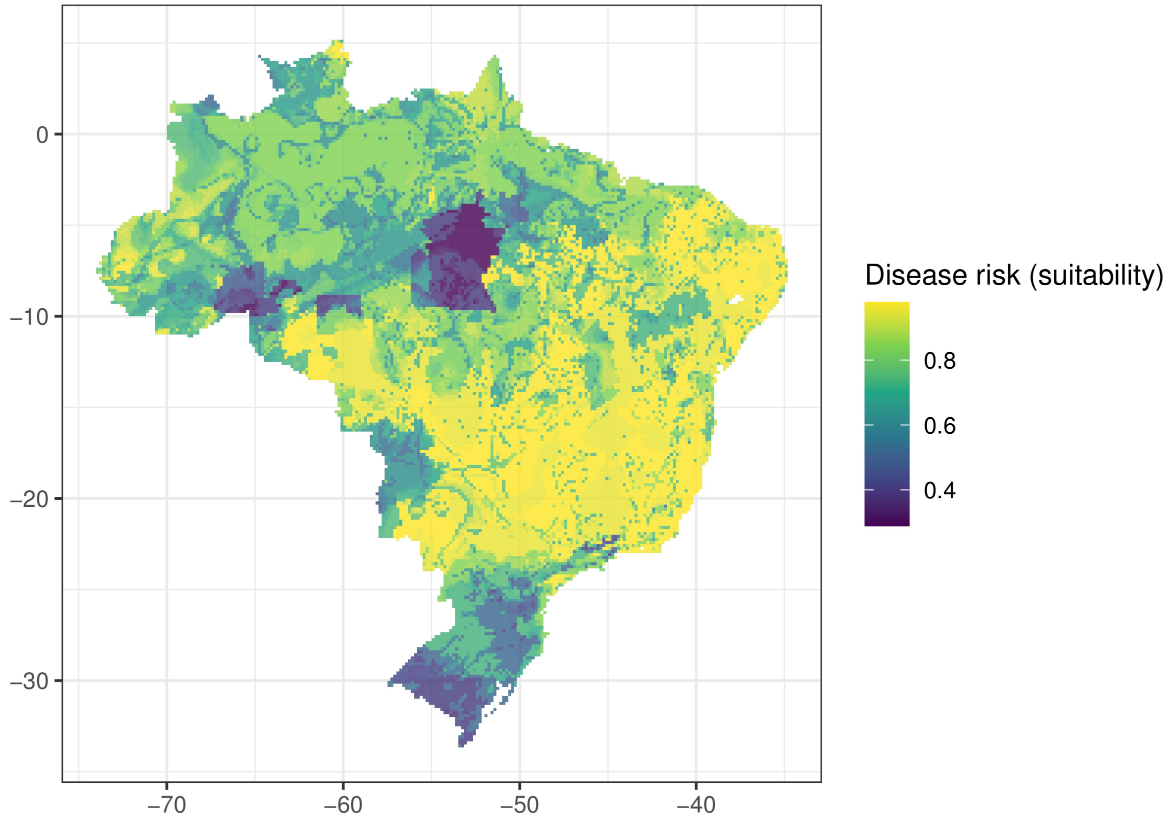


```r
dng_test22<-dng_test2+theme(panel.grid.major = element_blank(), panel.grid.minor = element_blank())
dng_test22
```

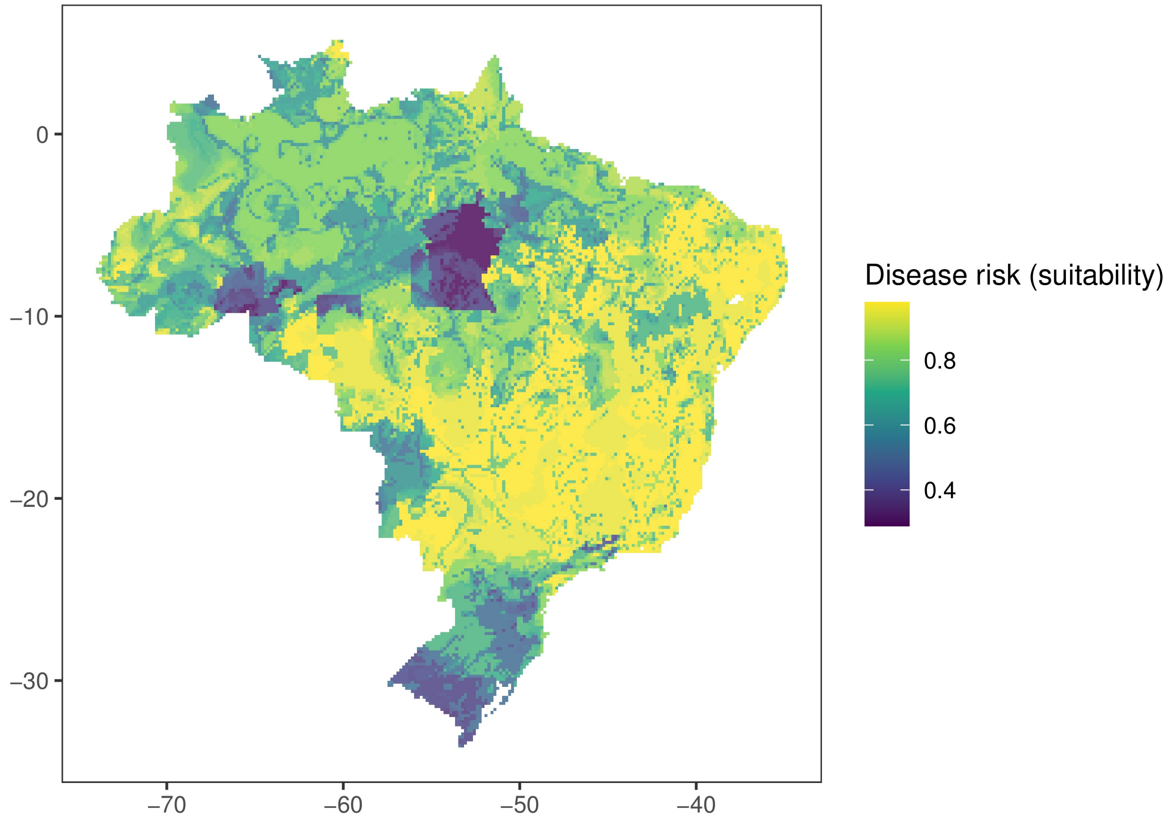

Now adding the occurrences as small red circles

```
dng_test22+geom_point(data = dng3,
            aes(x = longitude,
                y = latitude,
                color=sp_name),
            shape=1,
            size = 2)+
  scale_shape_manual(values = 1) +
  scale_color_manual(values = "#FC4E07")+
  labs(color="Disease Occurrences", shape=NULL)
```

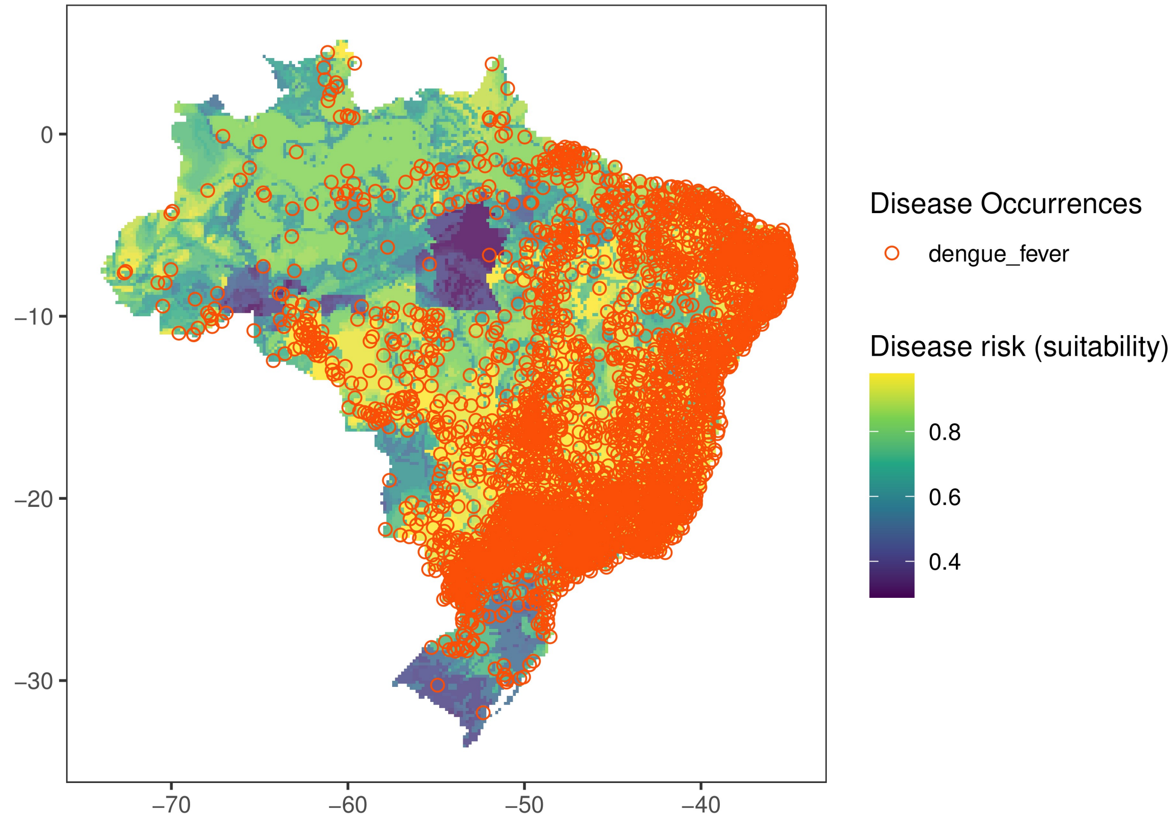

pROC metric for model comparasion

```
pROC_dng_clim <- pROC(continuous_mod=dng_clim_en2,
                      test_data = dng4,
                      n_iter=1000,E_percent=5,
                      boost_percent=50,
                      parallel=FALSE)
pROC_dng_clim$pROC_summary
```

```
##             Mean_AUC Mean_pAUC_ratio_at_5%               P_value
##            0.6934493             1.1093226             0.0000000
```

Now getting the response curves

```
rcurve(m_dng_clim)
```

```
## The id argument is not specified; The modelIDs of 45 successfully fitted models are assigned to id..
```

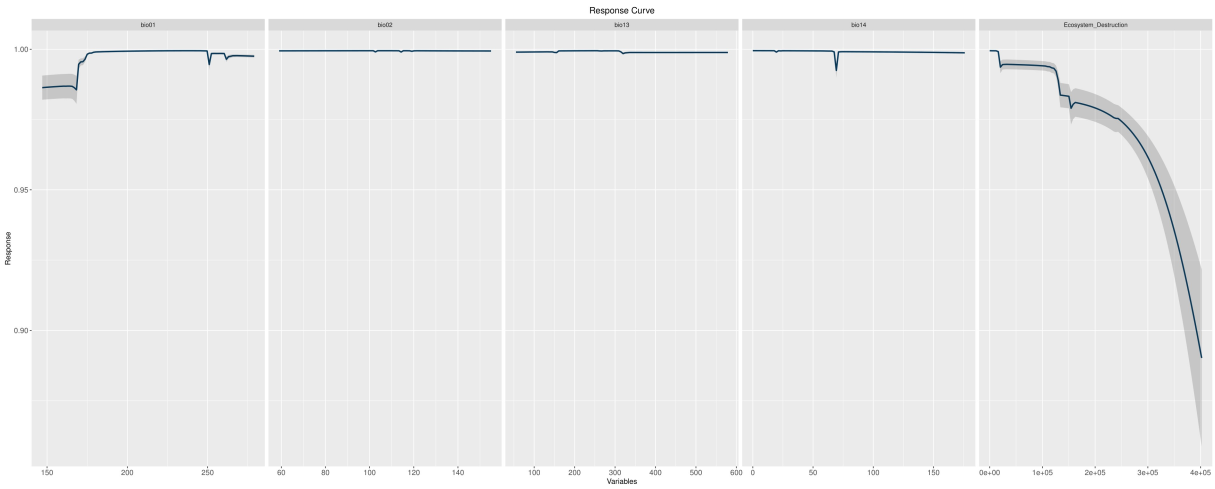

Now to get the relative variable importance, the variable contribution for the dengue environment only model:

```
plot(getVarImp(m_dng_clim))
```

```
##
## The variable importance for all the models are combined (averaged)...
```

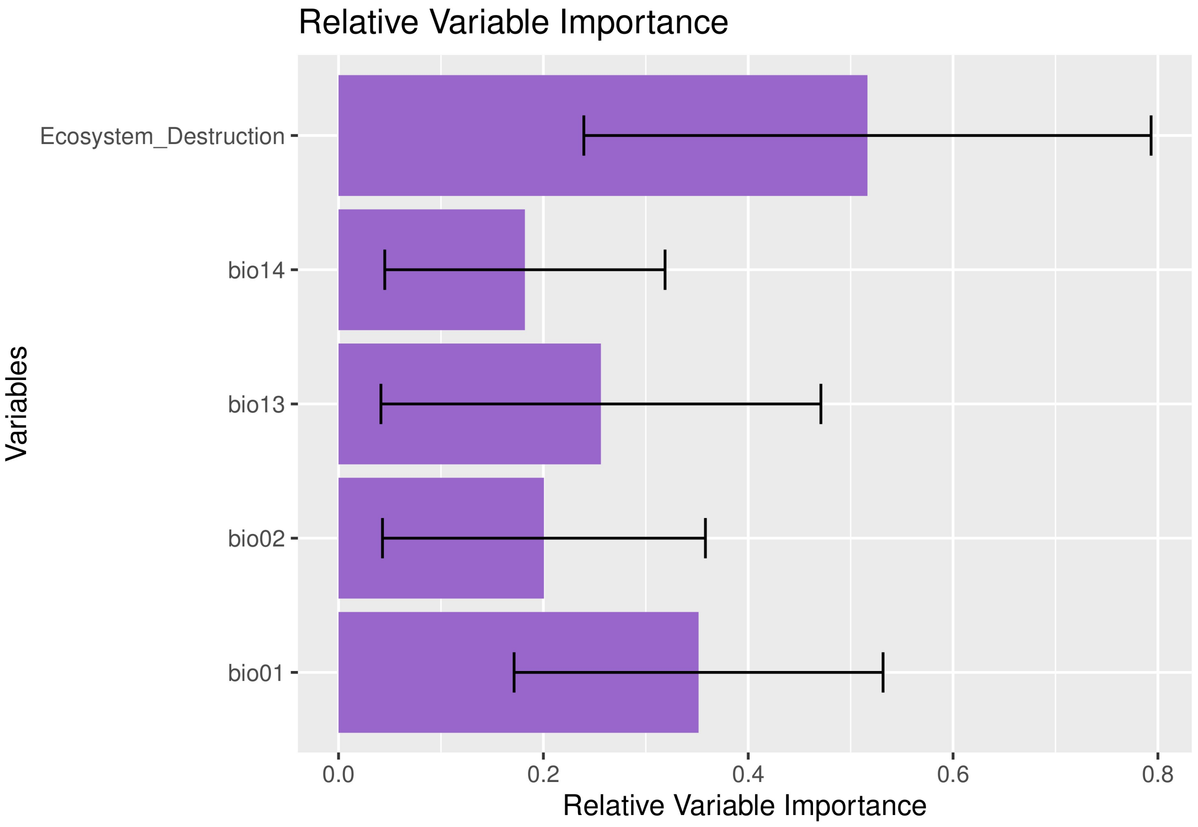

This is the end of the example with dengue fever. Any adtional doubts please sende email to arthurama.magalhaes@gmail.com Thank you in advance, Arthur
